# *UACA* locus is associated with breast cancer chemoresistance and survival

**DOI:** 10.1038/s41523-022-00401-5

**Published:** 2022-03-23

**Authors:** Qianqian Zhu, Emily Schultz, Jirong Long, Janise M. Roh, Emily Valice, Cecile A. Laurent, Kelly H. Radimer, Li Yan, Isaac J. Ergas, Warren Davis, Dilrini Ranatunga, Shipra Gandhi, Marilyn L. Kwan, Ping-Ping Bao, Wei Zheng, Xiao-Ou Shu, Christine Ambrosone, Song Yao, Lawrence H. Kushi

**Affiliations:** 1 Department of Biostatistics and Bioinformatics, Roswell Park Comprehensive Cancer Center, Buffalo, NY USA; 2 Division of Epidemiology, Department of Medicine, Vanderbilt Epidemiology Center, Vanderbilt-Ingram Cancer Center, Vanderbilt University Medical Center, Nashville, TN USA; 3 Division of Research, Kaiser Permanente Northern California, Oakland, CA USA; 4 Department of Cancer Prevention and Control, Roswell Park Comprehensive Cancer Center, Buffalo, NY USA; 5 Department of Medicine, Roswell Park Comprehensive Cancer Center, Buffalo, NY USA; 6 Shanghai Municipal Center for Disease Prevention and Control, Shanghai, China

**Keywords:** Genome-wide association studies, Chemotherapy

## Abstract

Few germline genetic variants have been robustly linked with breast cancer outcomes. We conducted trans-ethnic meta genome-wide association study (GWAS) of overall survival (OS) in 3973 breast cancer patients from the Pathways Study, one of the largest prospective breast cancer survivor cohorts. A locus spanning the *UACA* gene, a key regulator of tumor suppressor Par-4, was associated with OS in patients taking Par-4 dependent chemotherapies, including anthracyclines and anti-HER2 therapy, at a genome-wide significance level ($P = 1.27 \times 10^{ - 9}$). This association was confirmed in meta-analysis across four independent prospective breast cancer cohorts (combined hazard ratio = 1.84, $P = 1.28 \times 10^{ - 11}$). Transcriptome-wide association study revealed higher *UACA* gene expression was significantly associated with worse OS ($P = 4.68 \times 10^{ - 7}$). Our study identified the *UACA* locus as a genetic predictor of patient outcome following treatment with anthracyclines and/or anti-HER2 therapy, which may have clinical utility in formulating appropriate treatment strategies for breast cancer patients based on their genetic makeup.

## Introduction

The past decade has witnessed remarkable advances in our knowledge of the genetic architecture of breast cancer susceptibility from numerous genome-wide association studies (GWAS). To date, over 300 independent breast cancer risk variants that passed genome-wide significance ($P \, < \, 5 \times 10^{ - 8}$) have been identified^1,2^. In contrast, parallel efforts to discover genetic variants associated with breast cancer prognosis lag far behind, with only 11 GWAS published to date and only four loci reaching or approaching the canonical genome-wide significance^3–13^.

Several factors may have hindered the progress in GWAS of breast cancer prognosis. First, unlike breast cancer risk as a binary phenotype that is available from most cancer epidemiology studies and can be readily aggregated from multiple studies to a large sample size required for GWAS analysis, breast cancer prognosis is a phenotype that is more challenging to capture and requires long term follow-up efforts after diagnosis. Thus, breast cancer survival outcome data are available only from a limited number of studies, but even then, survival bias is a major concern in many of those studies. Second, heterogeneity in tumor subtype, histopathological presentation, treatment received, and comorbidities can have major effect on prognosis, which may have contributed to the lack of success in replicating loci across the prior GWAS of breast cancer prognosis. Detailed clinical annotation data are critical to control for potential confounding or interaction effects in GWAS of cancer prognosis, as well as to the meaningful interpretation of these findings. However, such data are often unavailable from studies where genotype data have been generated.

Despite these challenges, there is evidence supporting a strong genetic influence on breast cancer prognosis^14,15^. In animal studies, mammary tumor progression varies by genetic strain, and candidate germline polymorphisms are prognostic^16^. In human studies, familial concordance of breast cancer mortality was observed among mother–daughter and sister–sister pairs^17^, and the hereditary component in breast cancer prognosis appears to be independent of patient, tumor, and treatment variables^18^. Indeed, variants identified from GWAS of breast cancer susceptibility are rarely associated with prognosis^19,20^, indicating distinct genetic architecture underlying susceptibility and prognosis. The agnostic GWAS approaches are expected to discover new genes and biological mechanisms underlying breast cancer progression and drug response, to identify potential targets for treatment, and to provide clinically useful biomarkers for risk stratification and prediction of treatment outcomes.

In the present study, we report findings from GWAS of breast cancer prognosis in the context of the Pathways Study, one of the largest contemporary breast cancer survivor cohorts with rich patient outcome data through long-term active follow-up, and accompanying pathological, clinical, treatment, and epidemiological data. We sought to replicate our promising findings in three independent breast cancer cohorts, followed by meta-analysis to synthesize the results across race/ethnicity and cohorts, and lastly, a transcriptome-wide association study (TWAS) to further confirm the causal gene.

## Results

### Replication of prior GWAS findings

To date, 24 lead variants and one suggestive causal variant from 11 GWAS of breast cancer prognosis have been reported^3–13^, only four of which reached or approached genome-wide significance. We attempted to replicate these variants in the Pathway Study, by matching patient sub-population, survival outcomes, and covariates with the original study (Table 1). Only one of the 24 variants (rs421379, $P = 0.015$) was nominally significant ($P \le 0.05$) and had consistent direction of effect in our replication analysis as the prior GWAS. rs421379 on chr 5 was reported to associate with breast-cancer specific death (BCSD) in young (age ≤40 years) European patients^6^. The sample size and event number of this patient subpopulation were limited in our study (nine events out of 109 patients). When tested in European-descent patients of all adult ages, no association with BCSD was found ($P = 0.92$), in contrast to the previous GWAS^9^.

**Table 1:** Replication of prior GWAS findings in pathways.

Publication ID (Year)	GWAS variants					Original GWAS								Replication in pathways^ξ^						
	rsID	Chr	Position	Alleles (Ref/Alt)	Patient population		Events	*N*_total_	*N*_event_	*P*-value^¥^	HR	Effect allele	EAF	*N*_total_	*N*_event_	*P*-value	HR	Effect allele	EAF^§^	Rsq
31949161 (2020)	rs6990375	8	70,571,531	G/A	EUR, ER+		BCSD (censored at 10 years)	55,701	2854	**6.35E−09**				2404	107	0.76	0.96	A	0.294	Genotyped
31904872 (2020) ^€^	rs1401069	3	116,279,888	T/G	EAS, ER+		Death, recurrence	953	159	5.29E−07	>1	T		372	55	0.61	1.23	G	0.931	0.99456
	rs76462307	1	168,539,377	G/A						1.17E−06	2.01	A				0.17	1.5	A	0.155	0.92452
	rs1560409	11	121,160,060	C/G						2.17E−06	>1	C				0.34	0.83	G	0.614	0.98566
	rs2178052	6	46,541,231	G/A						5.47E−06	>1	A				2.69E−02	0.55	A	0.219	0.99824
	rs1024176^†^	1	168,526,103	G/A						2.43E−05	1.67	G				0.72	0.93	G	0.373	0.99947
30787463 (2019)	rs4717568	7	70,400,700	T/C	EUR, ER+		BCSD (censored at 15 years)	64,171	4116	1.28E−07	0.88	T	0.62	2404	113	0.29	0.86	T	0.412	0.99081
	rs67918676	7	27,445,956	A/AT	EUR, ER−			16,172	2125	1.38E−07	1.27	A	0.12	Absent in pathways						
	rs370332736	6	50,395,136	AACTT/A	EUR			96,661	7697	2.48E−07	1.16	A	0.09	Absent in pathways						
29158497 (2017)	rs715212	9	18,786,181	A/C	EUR, ≤40 years at diagnosis		Death, recurrence	2172	642	**5.37E−08**	1.38	C	0.27	67	12	0.16	2.03	C	0.258	0.98589
25890600 (2015)	rs2059614	11	125,259,424	A/G	EUR, ER-		BCSD (censored at 10 years)	6881	920	**1.30E−09**	1.90	G	0.06	393	48	0.53	1.26	G	0.073	Genotyped
	rs148760487	2	163,778,613	A/G	EUR			37,954	2900	**1.50E−08**	1.88	G	0.01	2798	155	0.92	0.95	G	0.013	0.97587
	rs7149859	14	68,066,489	G/T	EUR, ER+			23,059	1333	7.00E−07	1.22			2404	107	0.50	1.10	T	0.444	0.98762
25964295 (2015)	rs8113308	19	52,445,386	T/C	EUR, ER+ with endocrine treatment		BCSD (censored at 10 years)	3682		6.34E−07	1.69	C	0.152	2117	95	0.06	0.62	C	0.139	0.9974
25867717 (2015)	rs166870	15	80,069,762	T/C	EAS, HR+ HER2−		Recurrence, 2nd cancer	1902	159	2.88E−07	2.3	T	0.13, 0.87	306	59	0.11	1.45	T	0.165	0.97658
	rs10825036	10	55,266,231	T/G	EAS, HR− HER2−			554	100	3.54E−07	2.26	G	0.32, 0.29	37	3	NA	NA	G	0.298	0.98285
25526632 (2014)	rs421379	5	91,275,313	T/C	EUR		BCSD (censored at 10 years)	2756	758	1.10E−06	1.49	T	0.08	2797	155	0.92	1.02	T	0.069	Genotyped
	rs12358475	10	11,848,792	G/A						1.80E−06	0.75	A	0.23			0.85	1.02	A	0.241	Genotyped
	rs1728400	16	86,434,446	C/A						5.60E−06	1.25		0.38^‡^			0.38	0.90	A	0.451	Genotyped
23319801 (2013)	rs421379	5	91,275,313	T/C	EUR, ≤40 years at diagnosis		BCSD	2052	704	9.50E−07	1.61	T	0.05^‡^	109	9	1.45E−02	5.93	T	0.069	0.99962
	rs1387389	1	164,689,762	G/A						3.80E−06	1.28		0.36^‡^			0.85	0.91	A	0.313	Genotyped
	rs3884558	15	61,702,779	A/G						3.90E−06	1.46		0.07^‡^			NA	NA	G	0.910	Genotyped
	rs2774307	1	114,670,969	A/G						7.90E−06	1.30		0.26^‡^			0.17	2.36	G	0.742	0.93071
	rs3785982	17	9,090,224	C/T						7.90E−06	1.40		0.12^‡^			0.92	1.07	T	0.099	Genotyped
22232737 (2012)	rs3784099	14	68,749,927	G/A	EAS		Death	6110	719	1.17E−07	1.49	A	0.13	449	40	0.37	0.65	A	0.092	Genotyped
	rs9934948	16	73,439,355	C/T	EAS					5.75E−06	1.29	C	0.46			0.10	0.68	C	0.464	Genotyped
					EUR			1145	229	6.00E−03	3.27	C	0.84	2798	464	0.83	0.98	C	0.852	Genotyped

One GWAS study (PubMed ID: 20332263) was not included as no significant variant was identified in the study.

^¥^*P*-values at or close to genome-wide significance ($P < 5 \times 10^{ - 8}$) were in bold.

^ξ^Replication in Pathways matched the setting reported in the original GWAS, including survival outcomes, patient population, and adjusted covariates.

^§^The EAF was calculated in the whole Pathways EAS or EUR cohort.

^€^Although the risk alleles of the four GWAS variants were not given in the publication, we were able to identify them using the accompanying genotyping and clinical data provided by the publication (see "Methods" section).

^†^The causal variant claimed in the study for the chromosome 1 locus where the lead GWAS variant is rs76462307.

^‡^Only MAF was reported in the corresponding paper.

### GWAS of breast cancer prognosis in the Pathways Study

**Fig. 1:**
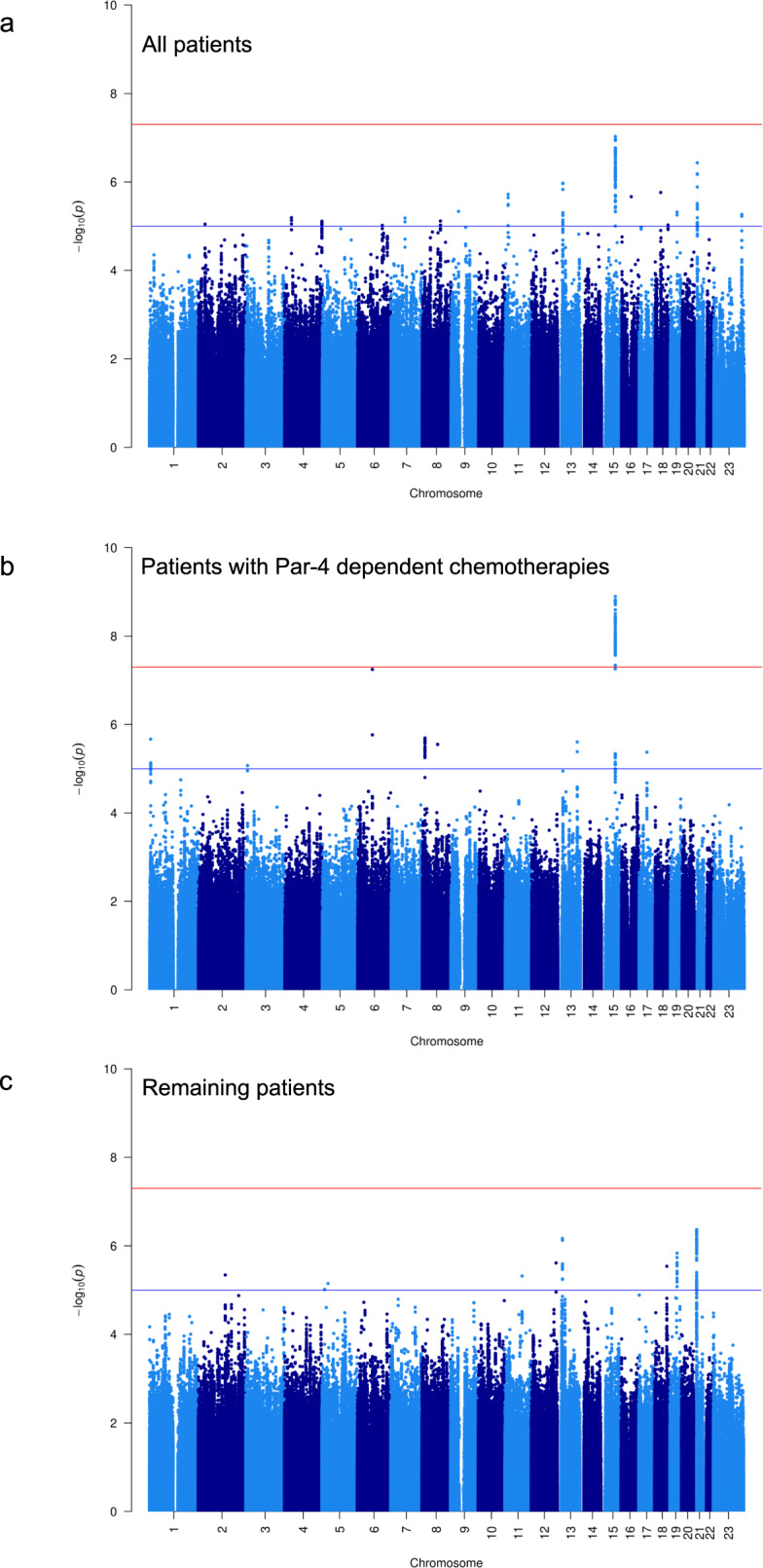
Manhattan plot for trans-ethnic meta-GWAS of OS in the Pathways Study. Analysis included all patients (**a**), patients taking Par-4 dependent chemotherapies (**b**), and the remaining patients (**c**) respectively.

In GWAS on OS within 2801 European-descent patients, the largest racial/ethnic group in the Pathways Study (Supplementary Table 1), no variants reached genome-wide significance. Clinical characteristics, including age at diagnosis, cancer grade and stage, hormonal receptor status, HER2 status, and treatment, were adjusted as covariates in the Cox regression model (see “Methods” section). The strongest association was on chromosomes 6 and 15 ($P = 3.63 \times 10^{ - 7}$ and $5.95 \times 10^{ - 7}$, respectively; Supplementary Fig. 1 and Supplementary Table 2). Trans-ethnic meta-GWAS across all populations (European, East Asian, Hispanic, and African) identified the same locus on chromosome 15 with close to genome-wide significance ($P = 9.42 \times 10^{ - 8}$ for rs11855431, Fig. 1 and Table 2 and Supplementary Figs. 2 and 3).

**Table 2:** The lead variant in trans-ethnic meta-GWAS of OS.

Cohort	Patient group*	Lead variant	Chr	Position	Alleles		MR-MEGA meta-regression					Pathways				Prospective GERA	
					Effect	Other	Effect direction	Meta *P*	*P*_HET-ANC_^†^	*P*_HET-RES_^‡^	Rsq	*P*-value^§^				Rsq	*P*-value
												EUR	EAS	HIS	AFR		
Pathways	All	rs11855431	15	71029751	C	T	+++−	9.42E−08	3.15E−03	0.54	0.99323	2.63E−05	1.64E−03	2.33E−02	0.36		
	Treatment	rs62019060	15	71051865	G	A	++++	1.27E−09	0.18	0.70	0.94063	9.82E−09	7.72E−03	0.37	0.33		
	Remaining	rs2747349	21	14741698	T	C	+++	4.28E−07	NA	0.06	0.71214	9.53E−07	NA	0.95	7.30E−03		
Pathways + Prospective GERA	Treatment	rs720251	15	70938445	T	C	+++++	5.49E−09	0.23	0.71	0.98588	4.19E−09	9.14E−03	0.48	0.94	0.98518	0.67
	Remaining	rs35759606	19	36582647	T	C	+−++	1.88E−06	2.55E−02	0.33	0.99046	7.84E−06	NA	0.95	4.20E−03	0.98141	0.52

*The “treatment” and “remaining” group correspond to patients received Par-4 dependent chemotherapies and the remaining patients respectively.

^†^*P*-value for heterogeneity correlated with ancestry.

^‡^*P*-value for residual heterogeneity.

^§^*P*-value from GWAS of OS within patients of European (EUR), East Asian (EAS), Hispanic (HIS), and African (AFR) population in the Pathways Study, respectively.

In analyses stratified by tumor ER status, an imputed variant passed genome-wide significance in GWAS within ER-positive (ER+) patients from Pathways European population (rs113113429 on chr 13, imputation Rsq = 0.91255, MAF = 0.053, $P = 2.89 \times 10^{ - 8}$; Supplementary Fig. 4). However, no variants in the locus were in high LD with rs113113429 (Supplementary Fig. 5), making it less likely a true hit. No genome-wide significant finding was observed in patients with ER-negative (ER−) cancer (top variant rs11690772 on chr 2, $P = 1.98 \times 10^{ - 7}$; Supplementary Fig. 6). Trans-ethnic meta-GWAS stratified by tumor ER status did not yield any genome-wide significance finding (lowest $P = 6.91 \times 10^{ - 7}$ and $1.67 \times 10^{ - 7}$ for ER+ and ER− patients, respectively; Supplementary Figs. 4 and 6),

### UACA locus affects patient outcomes after doxorubicin or anti-HER2 therapy

**Fig. 2:**
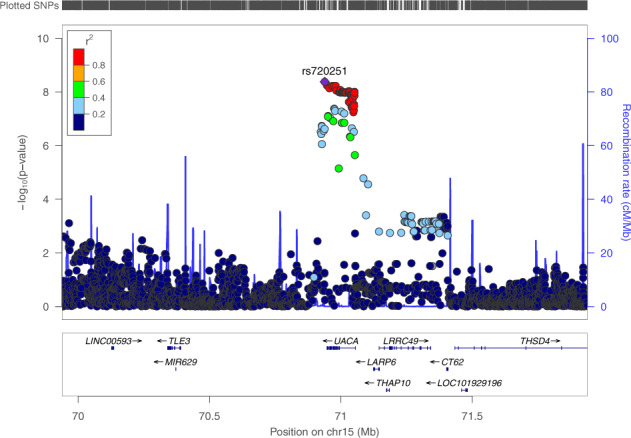
Locuszoom plot for the *UACA* locus in GWAS of OS within the Pathways European population when including patients taking Par-4 dependent chemotherapies. The 2 Mb region centered on the lead variant rs720251 was plotted. LD structure was based on 1000 genomes EUR population (Nov 2014).

**Fig. 3:**
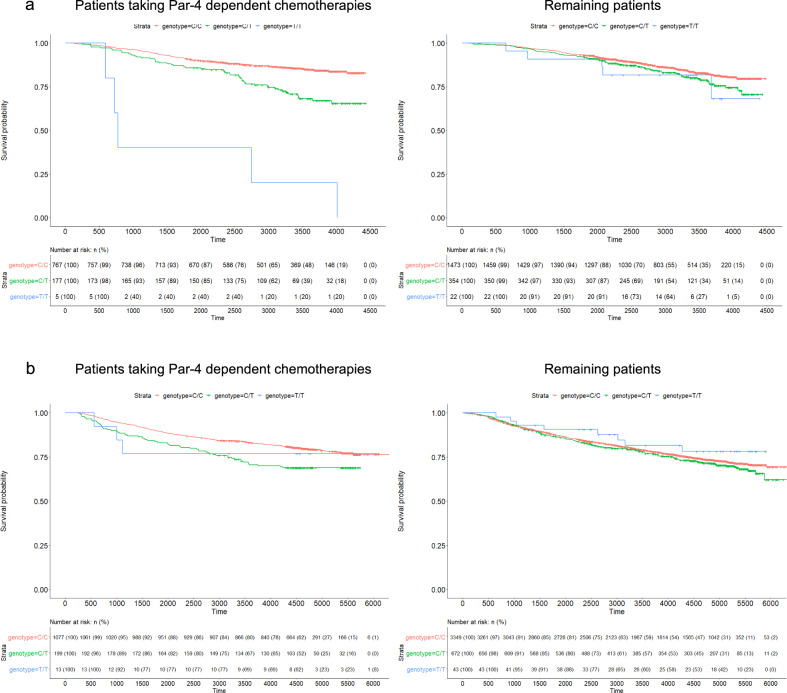
Kaplan–Meier plots of rs720251. KM plot in the Pathways European population (**a**), and the SBCSS + SBCS cohort (**b**). Patients were separated by whether they took Par-4 dependent chemotherapies. Patients with imputation dosage <0.5 were considered having genotype CC. Patients with imputation dosage ≥1.5 were considered having genotype TT. Patients with imputation dosage in-between were considered having genotype CT.

The top locus on chr 15 identified in our trans-ethnic meta-GWAS of OS contains the *UACA* gene, a key regulator of tumor suppressor Par-4^21^. Recent studies have suggested that Par-4 inhibition is involved in breast cancer recurrence and resistance to chemotherapy^22–24^. We thus tested the interaction between chemotherapy status and rs11855431, the lead variant of the *UACA* locus, for its effect on OS, and found the interaction was significant ($P = 6.1 \times 10^{ - 3}$). Since anti-HER2 agents and doxorubicin were shown to lead to Par-4-dependent multinucleation and cell death^22^, we further tested the interaction with these two Par-4 dependent agents vs. with all other chemotherapeutic agents. The results confirmed the interactive effect on OS to be specific to anti-HER2 and doxorubicin therapies ($P = 2.4 \times 10^{ - 4}$ vs. $P = 0.80$ for all other chemo treatment). Subsequent GWAS performed separately in these two treatment groups within the European population (Supplementary Figs. 7 and 8 and Supplementary Table 2) revealed that *UACA* was the only locus associated with OS at genome-wide significance in patients taking Par-4 dependent chemotherapies (Hazard ratio (HR) = 2.79, $P = 4.19 \times 10^{ - 9}$ for the minor allele (T) of lead variant rs720251; Figs. 2 and 3), with no effect in the remaining patients (HR = 1.20, $P = 0.19$; Fig. 3 and Supplementary Fig. 7). The association remained significant in trans-ethnic meta-GWAS within patients taking Par-4 dependent chemotherapies ($P = 1.27 \times 10^{ - 9}$ for the lead variant rs62019060), where the G allele of rs62019060 was consistently associated with poorer patient survival in all four populations (HR = 3.01, 3.19, 1.90, 1.51, and allele frequency = 0.101, 0.099, 0.092, 0.354 in European, East Asian, Hispanic, African population, respectively, Fig. 1 and Table 2, and Supplementary Fig. 9). In contrast, no association was observed for rs62019060 in trans-ethnic meta-GWAS of the remaining patients ($P = 0.21$). The three lead variants, rs62019060, rs720251, and rs11855431 are in high linkage disequilibrium (LD) in the 1000 Genomes EUR population (all pairwise *r*^2^ ≥ 0.90, see “Methods” section). No other genes involving in the UACA-Par-4 pathway associated with OS in patients taking Par-4 dependent chemotherapies (Supplementary Fig. 10).

**Fig. 4:**
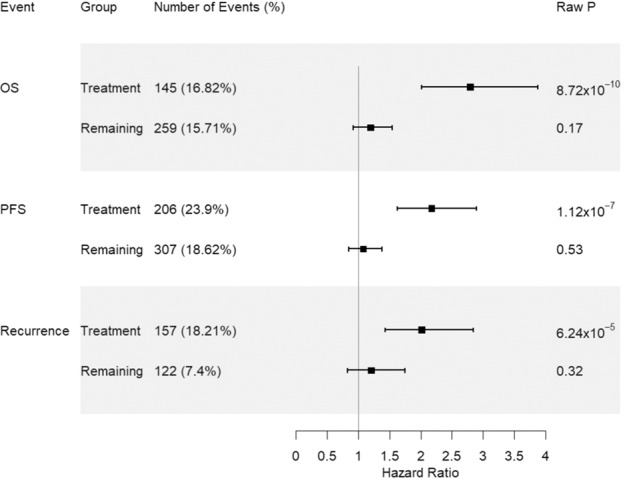
Association of rs720251 with OS, PFS, and breast cancer recurrence in the Pathways European population. The “treatment” and “remaining” group correspond to patients received Par-4 dependent chemotherapies and the remaining patients, respectively. The estimated hazard ratio as well as its 95% confidence interval were plotted. Raw *P*-values from the corresponding cox models were given for direct comparison across outcomes.

Because Par-4 has been reported to play an important role in breast cancer recurrence^22–24^, we then evaluated the impact of *UACA* locus on recurrence and progression-free survival (PFS), which includes both recurrence and death, among patients treated with anti-HER2 or doxorubicin chemotherapies. Although rs720251, the lead variant in European population, was associated with both recurrence (HR = 2.02, raw $P = 6.24 \times 10^{ - 5}$, Fig. 4) and PFS (HR = 2.17, raw $P = 1.12 \times 10^{ - 7}$; Fig. 4), the associations were not as strong as with OS despite using the same patients. As after cancer diagnosis, patients can progress to death through two mutually exclusive courses: death after recurrence versus death without recurrence, we used a competing risk model to investigate how the *UACA* locus may affect these two courses. rs720251 was observed to associate with both death after recurrence (subdistribution hazard ratio (SHR) = 2.59, $P = 7.7 \times 10^{ - 5}$) and death without recurrence (SHR = 2.23, $P = 1.1 \times 10^{ - 2}$), suggesting a more general effect beyond recurrence. In addition, we found rs720251 associated with both breast cancer-specific death (SHR = 2.49, $P = 1.3 \times 10^{ - 6}$) and other cause of death (SHR = 2.79, $P = 3.9 \times 10^{ - 2}$). As cardiotoxicity is a well-recognized adverse effect of both anti-HER2 or doxorubicin chemotherapies^25–27^, we then investigated whether the *UCAC* locus also affected cardiotoxicity in patients received those treatments using a competing risk model. Indeed, we found rs720251 associated with both death due to cardiovascular disease (SHR = 2.80, $P = 7.7 \times 10^{ - 3}$) and other cause of death (SHR = 2.61, $P = 2.6 \times 10^{ - 6}$).

### Validation in patients of European descent in the Genetic Epidemiology Research on Aging (GERA) Cohort

The GERA breast cancer cohort included both a prospective component, which involved 880 incident cases who had breast cancer diagnosis after collection of biospecimens for genotyping, and a retrospective component, which involved 1983 prevalent cases who had cancer diagnosis before biospecimen collection. Of the 880 incident cases, 158 (18%) received anti-HER2 or doxorubicin therapies, in comparison to 34% in the Pathways Study. We observed a non-significant yet consistent trend that *UACA* locus was associated with OS in patients treated with the Par4-dependent agents (HR = 1.61, $P = 0.67$ for the minor allele T of rs720251); yet no effect was seen in the remaining patients (HR = 1.13, $P = 0.71$).

**Fig. 5:**
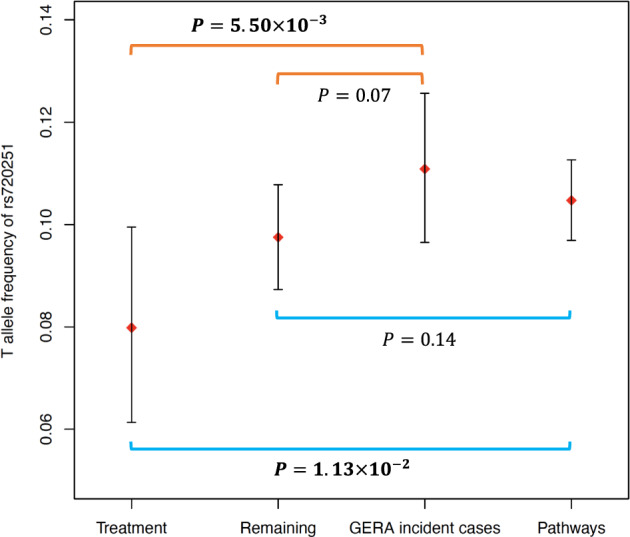
Comparison of risk allele (T) frequency of rs720251 in the GERA prevalent cases with its population frequency. The “treatment” and “remaining” group correspond to the GERA prevalent patients with and without Par-4 dependent chemotherapies respectively. T allele frequency at population level was estimated using the GERA incident cases or using all European-descent patients from the Pathways Study (“Pathways”). The estimated T allele frequency as well as its 95% confidence interval from 10,000 bootstrap samples were plotted. The *P*-values were calculated by comparing the T allele frequency between the two corresponding groups connected by the brackets in the plot from the 10,000 bootstrap replications. Significant *P*-values (≤0.05) were in bold.

Based on the hypothesis that the T allele of rs720251 was associated with higher risk of death in patients treated with Par-4 dependent chemotherapies, we expect that the prevalent cases who were treated with these agents represent a survival bias and thus carry T alleles at a lower frequency than that in the incident cases, but no such difference should be expected in cases without such treatment. Indeed, this was what we observed between prevalent and incident cases (Par4-dependent treatment: 7.98% vs. 11.08%, bootstrap $P = 5.50 \times 10^{ - 3}$; other treatment: 9.75% vs. 11.08%, bootstrap $P = 0.07$; Fig. 5, see “Methods” section).

### Validation in patients of European descent in the Data Bank and Biorepository (DBBR) cohort

The DBBR cohort included 451 breast cancer patients of European descent treated with anti-HER2 or doxorubicin therapies at Roswell Park Comprehensive Cancer Center. In comparison to the Pathways Study, the DBBR cohort were younger (mean age at diagnosis: 51 vs. 55.7 years) and more likely to receive radiation therapy (82.5% vs. 23.1%) (Supplementary Table 3). None of the four top variants in the *UACA* locus was significantly associated with OS in this cohort (lowest $P = 0.56$ for rs11855431, Supplementary Table 4).

### Validation in patients of East Asian descent in the Shanghai Breast Cancer Survival Study (SBCSS) and Shanghai Breast Cancer Study (SBCS)

**Fig. 6:**
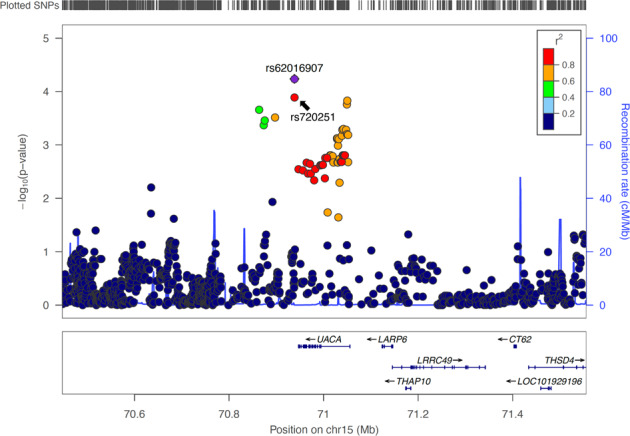
Locuszoom plot for the association between OS and the *UACA* locus within the SBCSS and SBCS patients taking anthracyclines. All evaluated variants are within 500 kb of the *UACA* gene. LD structure was based on 1000 genomes ASN population (Nov 2014).

The two Shanghai studies pre-dated the advent of anti-HER2 therapy, and thus very few patients received this treatment. While doxorubicin was the dominant anthracycline agent used in the three US cohorts, >96% of the 1289 SBCSS and SBCS patients treated with anthracycline-containing regimens received epirubicin or pirarubicin. We evaluated 1297 variants within 500 kb around *UACA* gene for their associations with OS stratified by anthracycline treatment. The most significant variant was rs62016907 (HR = 1.72, $P = 5.76 \times 10^{ - 5}$, imputation Rsq = 1.00, Fig. 6) in patients with anthracycline treatment, while no association was found for this SNP (HR = 1.06, $P = 0.48$) among patients receiving no anthracycline treatment. Notably, rs62016907 was genotyped in the Pathways Study and also associated with OS among patients of East Asian population who received Par-4 dependent chemotherapies (HR = 3.00, $P = 8.68 \times 10^{ - 3}$). The lead variant in patients of European population in the Pathways Study, rs720251, was the second most significant variant associated with OS in the SBCSS and SBCS patients treated with anthracyclines (HR = 1.67, $P = 1.29 \times 10^{ - 4}$, imputation Rsq = 0.92). Again, no association was found between rs720251 and OS among patients without anthracycline treatment (HR = 1.06, $P = 0.41$).

### Meta-analysis of rs720251 in the UACA locus across all four cohorts

**Fig. 7:**
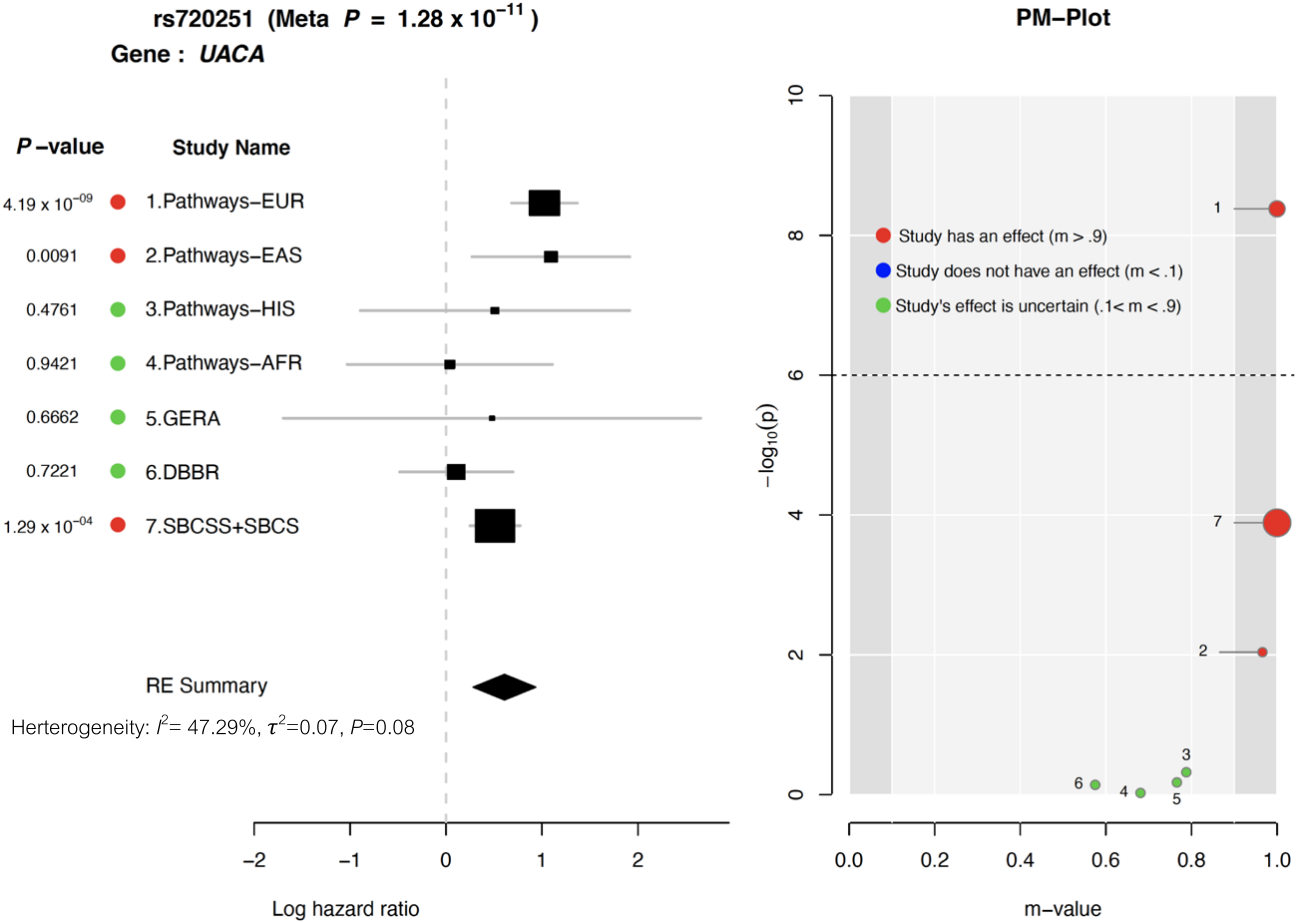
Meta-analysis of rs720251 across all four independent prospective cohorts evaluated in this study. Pathways-EUR, Pathways-EAS, Pathways-HIS, and Pathways-AFR represents the European, East Asian, Hispanic, and African population of the Pathways Study respectively. GERA corresponds to the incident cases of European descent from the GERA cohort. SBCSS + SBCS represents the SBCSS and SBCS patients. Meta *P*-value was calculated using METASOFT based on Han and Eskin’s Random Effects model. The forest plot (left) displays *P*-value, study name, log hazard ratio and its standard error from the cox regression model of the corresponding study. The PM-Plot (right) displays the *m*-value (the posterior probability that the effect exists) of each study along with its *P*-value. The size of the symbol represents the study sample size.

In meta-analysis involving 3359 patients (652 events) treated with Par-4 dependent chemotherapies across the above four prospective cohorts using Han and Eskin’s Random Effects model^28^, the T allele of rs720251 was significantly associated with OS (combined HR = 1.84, $P = 1.28 \times 10^{ - 11}$, Fig. 7). Posterior probability calculated from the meta-analysis suggested a genetic effect of this variant in the European and East Asian populations of the Pathways Study, as well as in the SBCSS and SBCS patients (*m*-value > 0.9).

### Higher UACA gene expression associated with worse breast cancer survival

**Fig. 8:**
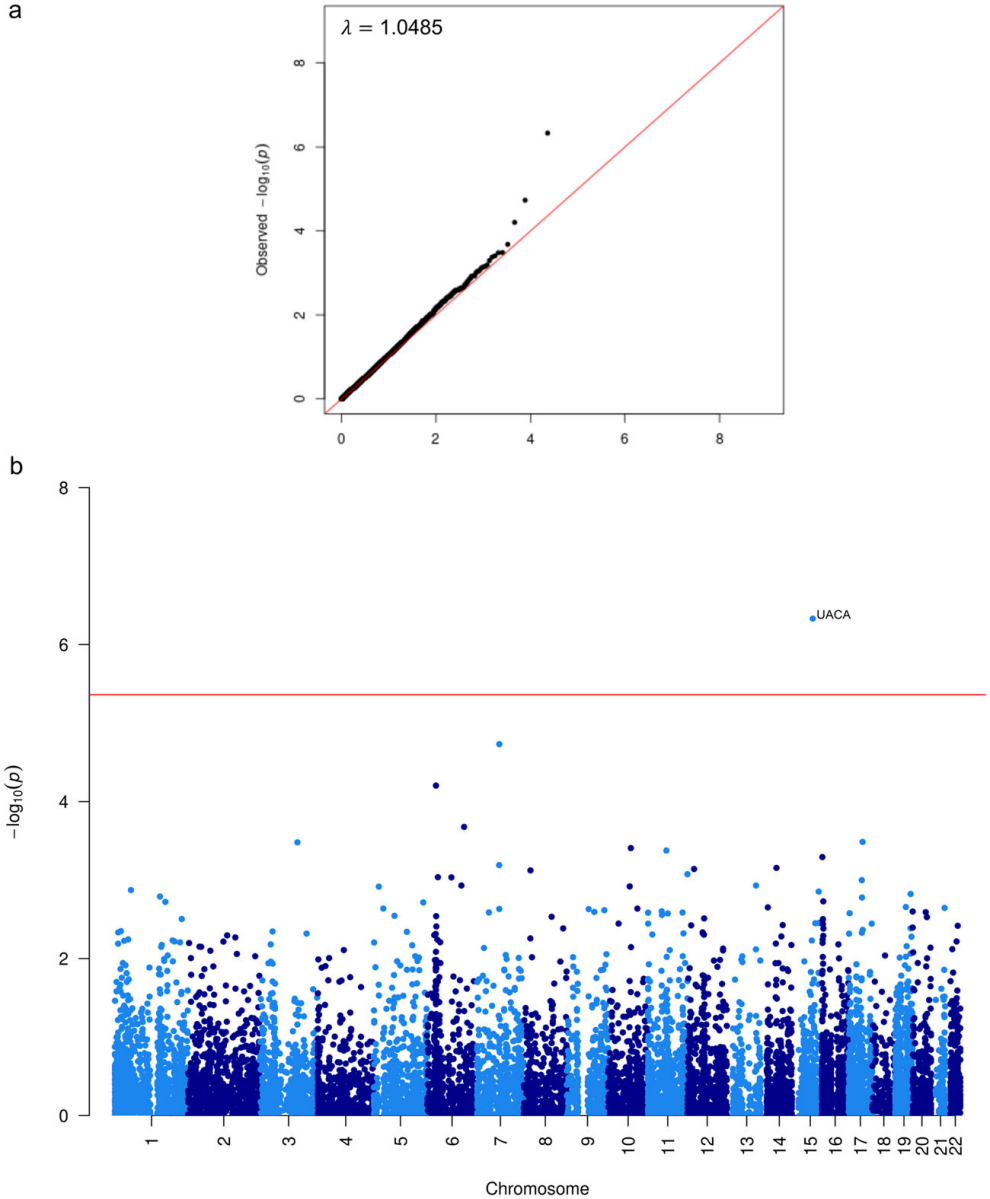
QQ plot and Manhattan plot for TWAS of OS in the Pathways European population when including only patients taking Par-4 dependent chemotherapies. **a** QQplot. **b** Manhattan plot. The red line in the Manhattan plot corresponds to transcriptome-wide significance (4.34 × 10^−6^) after multiple hypothesis correction for 11,520 genes.

To confirm that the *UACA* gene is indeed responsible for the GWAS association and to infer the underlying biological mechanism, we interrogated cis-expression quantitative trait locus (eQTLs) from a meta-analysis of 31,684 blood samples collated by the eQTLGen consortium^29^. Among genes that are 1 Mb nearby, the lead variant in the trans-ethnic meta-GWAS, rs62019060, was identified as an eQTL for only *UACA*, with the risk allele G associated with higher expression (*Z*-score = 20.57, Bonferroni corrected $P = 6.10 \times 10^{ - 86}$). Furthermore, of the 49 tissues tested for the GTEx eQTL analysis^30^, rs62019060 is a significant eQTL for *UACA* in 17 tissues (multi-tissue meta-analysis $P = 3.70 \times 10^{ - 124}$, Supplementary Fig. 11). The risk G allele was consistently associated with higher *UACA* expression across tissues, including breast (Supplementary Fig. 12). In a transcriptome-wide association study (TWAS) of OS among patients of European population treated with Par-4 dependent chemotherapies in the Pathways Study, *UACA* expression was significantly associated with higher risk of death (HR = 11.38, $P = 4.68 \times 10^{ - 7}$). It was also the only gene reaching transcriptome-wide significance (Fig. 8).

## Discussion

Breast cancer survival has proven to be a challenging phenotype for GWAS to identify bona fide germline genetic variants that are either prognostic regardless of treatment, or predictive for the efficacy of specific treatment. The goal of the former is rooted in tumor biology, whereas the latter falls into the area of pharmacogenomics and potentially useful for optimizing treatment with specific regimens. To date, most prior GWAS on breast cancer survival were conducted for prognostication, either with all patients combined or stratified by tumor ER status. This is likely due to the fact that many prior analyses were conducted by pooling data from patient populations originally genotyped for analysis of cancer risk, where detailed treatment data were often lacking. Nonetheless, findings from these prior GWAS suffer from low reproducibility. The 11 prior GWAS of breast cancer survival have reported 24 loci with four loci at or close to genome-wide significance (*P* < 5 × 10^−8^). However, none of them could be replicated by others. In fact, in three separate analysis of breast cancer-specific death conducted by the same group based on different yet overlapping patient populations, no replicable variants emerged^5,11,12^. It is thus not surprising that our evaluation of the prior GWAS variants in the Pathways Study could not replicate any with high level of confidence.

Failure in replicating prior GWAS findings for breast cancer prognosis along with no genome-wide significant finding in our initial GWAS within all, ER+, or ER− patients highlights the difficulty in identifying prognostic genetic markers applicable to all patients or to patients of certain subtypes, considering breast cancer is intrinsically heterogeneous. Aside from the heterogeneities in patient populations, tumor subtypes, duration of follow-up, and survival endpoints tested, limited sample size and power is often cited as another major challenge. However, the largest GWAS to date for breast cancer survival included >96,000 patients and close to 8000 events, yet no variants reached genome-wide significance^11^, whereas prior GWAS for breast cancer risk with a sample size of this magnitude produced many highly replicable hits with modest effect sizes. An important factor to be contemplated that might help explain the conundrum is cancer treatment, which has a major impact on patient outcomes after cancer diagnosis. There is great diversity in treatment modalities, from different surgical procedures to radiation therapy, systemic chemotherapy, or endocrine therapy in the neoadjuvant or adjuvant setting. Each patient’s course of treatment is likely unique and subject to the influence of physician discretion, personal preference, and psychosocial factors. The sheer magnitude of treatment heterogeneities might overwhelm the modest genetic effect, leading to spurious findings difficult to replicate when data structure changes in a separate analysis. It is thus critical to carefully consider patient treatment while conducting GWAS for breast cancer survival.

Based on almost 4000 breast cancer patients in the Pathways Study with detailed treatment data, our analysis began with a GWAS of OS in all patients while controlling for cancer treatment. The top variant located in a locus containing *UACA* was close to genome-wide significance cutoff. Strong biological evidence supports the role of *UACA* in helping cancer cells escape Par-4 induced apoptosis by preventing trafficking of Par-4 receptor, GRP78, to the cancer cell surface^21^, whereas Par-4-dependent cancer cell multinucleation and cell death was shown indispensable for the anti-cancer activities of anti-HER2 agents and anthracyclines^22^. Subsequent analyses stratified by Par-4-dependent therapies vs. other therapies confirmed the association to be specific to patients who received anti-HER2 and/or anthracycline-containing therapies. Meta-analysis across four independent cohorts confirmed the association with a strong per allele HR of 1.84 for the top variant rs720251. Our eQTL analysis and TWAS further supported the robustness of this finding. We identified a pharmacogenomic marker for anthracyclines and HER2 targeted therapy, two commonly used anti-breast cancer agents. Considering the risk allele being rather common, the observed large effect size, and the consistent effect across diverse populations (MAF > 9% and HR > 1.5), particularly the effect on the East Asian population was independently validated, a genetic test on the *UACA* locus may bring broad clinical relevance in guiding physicians’ decisions on the use of these agents. In addition, our finding suggests that patients carrying the germline risk alleles likely have higher *UACA* expression in both normal cells and breast tumor cells. Higher *UACA* expression in tumor cells results in a reduced sensitivity to therapeutic agents that rely on Par-4 induced apoptosis and hence worse prognosis. In normal cells, it has been shown that activation of p53 in normal cells, e.g., following doxorubicin treatment, promotes normal cells secreting Par-4 to induce tumor cell apoptosis^31^. However, being a principal binding partner of Par-4, UACA can sequester Par-4 inside normal cells and suppress Par-4 secretion^31^. Therefore, higher *UACA* expression in normal cells results in lower extracellular Par-4 level, which again leads to reduced tumor apoptosis and worse prognosis. Such discoveries highlight the potential of targeting the Par-4 pathway as a novel therapeutic approach for improving outcomes of breast cancer^24,32,33^.

In conclusion, in a large GWAS of breast cancer survival, we identified a genome-wide significant genetic predictor for the efficacy of two widely used breast cancer agents, namely anti-HER2 therapy and anthracyclines. Upon future studies to further validate our findings and to evaluate the clinical utility, we anticipate that the knowledge of the *UACA* genotypes or expression level would help physicians identify patients who are most likely to benefit from anthracyclines and HER2 targeted therapy, and provide complementary or alternative treatment to those who are not.

## Methods

### Study population

The Pathways Study is a prospective cohort study of a diverse population of recently diagnosed breast cancer survivors in Kaiser Permanente Northern California (KPNC)^34^. Recruitment into the cohort was from January 2006 to May 2013. Eligibility criteria were: age ≥ 21 years; current KPNC member; recently diagnosed with invasive breast cancer; no prior history of other invasive cancer other than non-melanoma skin cancer; primary language of English, Spanish, Cantonese, or Mandarin; and lived within a 65-mile radius of a field interviewer. Blood and saliva specimens were collected around the time of enrollment, which was on average less than two months after pathology-confirmed diagnosis. Recurrences were identified through self-report, the KPNC Cancer Registry, or through an algorithm designed to identify possible recurrences through clinical events, such as ICD9 or 10 code, suggesting a recurrence or re-initiation of chemotherapy. All recurrences were confirmed through medical record review.

The Kaiser Permanente Genetic Epidemiology Research on Aging (GERA) Cohort consists of a diverse cohort of more than 100,000 adults who are members of KPNC, and participants in its Research Program on Genes, Environment and Health (RPGEH), which started in 2005. The GERA cohort only included RPGEH participants who had answered a detailed survey in 2007, provided saliva samples for extraction of DNA since July 2008, and given broad consent for the use of their data in studies of health and disease. Our study selected breast cancer patients from the GERA cohort who were successfully genotyped and were not participants of the Pathways Study. The GERA cohort included incident breast cancer cases, who were diagnosed after their saliva samples were collected, as well as prevalent breast cancer cases, who were diagnosed before saliva sample collection.

For both the Pathways Study and GERA cohort, detailed information on cancer diagnosis and treatment were obtained from KPNC electronic health records, including the KPNC Cancer Registry. Cohort members were followed for mortality through the KPNC mortality linkage file, which incorporates information from various sources, including medical system records, Social Security Administration databases, and the National Death Index. In the Pathways Study, deaths reported to us by family members were also included.

The Data Bank and Biorepository (DBBR) cohort included 451 female breast cancer patients selected from Roswell Park Comprehensive Cancer Center Data Bank and Biorepository^35^. All patients were self-reported white and received either Doxorubicin or HER2 targeted therapy.

The Shanghai Breast Cancer Survival Study (SBCSS) was conducted in urban Shanghai and recruited 5042 patients with breast cancer between March 2002 and April 2006 (participation rate: 80.1%); 98% of patients provided an exfoliated buccal cell sample^36,37^. The Shanghai Breast Cancer Study (SBCS) is a population-based case–control study that recruited incident patients with breast cancer from urban Shanghai between August 1996 and March 1998 and between April 2002 and February 2005^36^. A total of 3448 patients were recruited (participation rate: 86.7%); 90.6% of participants provided a blood or exfoliated buccal cell sample. Medical charts were reviewed to verify cancer diagnosis and obtain clinical information such as cancer stage, tumor ER and progesterone receptor (PR) status, and primary treatments (surgery/mastectomy, radiation therapy, chemotherapy). The SBCSS also collected detailed information on treatment regimens. Patients with cancer have been followed for survival status and breast cancer recurrence through regular record linkages with the Shanghai Vital Statistics Registry. In the SBCSS, in-person surveys at 18-month, 3, 5, and 10 years after diagnosis were also carried out to update exposure information and collect information on cancer recurrence. Because of a time overlap during recruitment, 1469 women participated in both the SBCSS and SBCS. For these overlapping patients, information from the SBCSS patients were used in the current study. We excluded patients with stage 0 disease, having no genetic information or no information on survival status from the study. A total of 5495 patients were included this study.

All study participants provided written informed consent before participating in the study and the Institutional Review Boards of all institutes involved approved the study protocols. Our study is compliant with the “Guidance of the Ministry of Science and Technology (MOST) for the Review and Approval of Human Genetic Resources”.

### Genotyping, quality control, and imputation

Genotyping of the Pathways Study was performed by Johns Hopkins Center for Inherited Disease Research (CIDR) using the Illumina Multi-Ethnic Global Array with inclusion of custom content from the BioVU breast cancer SNP subset. A total of 4480 samples from 4376 patients were successfully genotyped and passed CIDR quality control (QC), where samples were evaluated by sample missing rate, gender mismatch, sex chromosome abnormalities, sample relatedness, and population structure. CIDR’s QC also resulted in variant removal if the variant missing rate was >2%, if there was more than one Mendelian error in the HapMap trios included in genotyping, if variants violated Hardy–Weinberg equilibrium (*P* < 1 × 10^−4^) or were discordant between duplicate samples (>1 discordant call) in 97 study duplicates. Positional duplicated variants were also removed. CIDR then took the remaining samples and variants after QC for imputation using the University of Michigan Imputation Server^38^ and the Haplotype Reference Consortium (HRC) reference panel^39^. Eagle2 was used for pre-phasing while miminac3 and minimac4 was used for imputation on autosomes and X chromosome respectively. Variant with imputation quality Rsq < 0.3 were excluded from analysis.

As the Pathways Study is a multi-ethnic cohort, we further separated the 4480 samples that passed CIDR QC into four populations (European, Asian, Hispanic, and African) based on self-reported race and ethnicity, and performed sample-level QC within each population. Samples were removed if the missing rate was >5%, the typed and reported sex did not match, there were abnormal inbreeding coefficients, or cryptic relatedness. Duplicate samples with higher sample missing rate were filtered out and population outliers were removed by using EIGENSTRAT^40^. EIGENSTRAT separated the Asian population into two clusters: East Asian and South Asian. The 72 samples of South Asian descent were excluded from further analysis due to limited sample size. At the end 2801, 450, 392, and 330 samples within European, East Asian, Hispanic, and African population were kept for further analysis.

The GERA cohort was genotyped using four Affymetrix Axiom arrays custom-designed for individuals of Non-Hispanic White (EUR), East Asian (EAS), African-American (AFR), and Latino (LAT) race/ethnicity^41,42^. Samples were assigned to different arrays based on their self-reported race/ethnicity. As only limited number of minority breast cancer patients were included in the GERA cohort, our analyses focused on European-descent patients genotyped using EUR array. As some EUR arrays were run at a time when Affymetrix had completely upgraded its reagent protocol from Kit A to Kit O, we included reagent kit as a covariate in our Cox regression models of the GERA cohort. We performed both sample-level and variant-level QC on these samples. In sample-level QC, samples were removed if the missing rate was >5%, the typed and reported sex did not match, there were abnormal inbreeding coefficients, or cryptic relatedness. Population outliers were removed using EIGENSTRAT. In variant-level QC, variants were removed if the missing rate was >2%, if there was more than one Mendelian error in HapMap trios included in genotyping, if variants violated Hardy–Weinberg equilibrium (*P* ≤ 1 × 10^−6^). Monomorphic variants were also excluded before imputation. Similar to the Pathways Study, we performed imputation using the University of Michigan Imputation Server and the HRC reference panel. Eagle2 and miminac3 were used for pre-phasing and imputation respectively on autosomes, while ShapeIT and minimac4 were used for pre-phasing and imputation respectively on X chromosome. Variant with imputation quality Rsq < 0.3 were excluded from analysis. Ultimately, 2909 samples within the European ancestry population were kept for analysis, including 880 incident cases and 1983 prevalent cases.

For replication in the DBBR cohort, we selected five variants (rs11855431, rs720251, rs62019060, rs6494889, and rs28607477) for genotyping using Applied Biosystems TaqMan SNP Genotyping assays. rs6494889 and rs28607477 were highly correlated with the three lead variants (rs11855431, rs62019060, and rs720251) in the Pathways EUR cohort (*R*^2^ of imputation dosage >0.94). rs62019060 failed genotyping and only the four remaining variants were used for association analysis.

The SBCSS and SBCS samples were genotyped primarily using one of the Affymetrix SNP 6.0 array, Illumina OncoArray, Illumina MEGA, or Illumina iCOGS platforms. Stringent criteria were used for QC for each dataset^43^. QC procedure include: samples were excluded if they (i) had genotyping call rate <95%; (ii) were male based on genotype data; (ii) had a close relationship with a Pi-HAT estimate >0.25; (iii) were heterozygosity outliers; (iv) were ancestry outliers. SNPs were excluded if they had (i) a call rate <95%; (ii) no clear genotyping clusters; (iii) a minor allele frequency <0.001; (iv) a Hardy–Weinberg equilibrium test of *P* < 1 × 10^−6^; (v) genotyping concordance <95% among the duplicated QC samples. The cleaned data were imputed using 1000 Genomes as reference. Details on the methodology of the parent studies have been described previously^36,37^. Only variants with MAF > 5% and Rsq $\ge$0.3 were included in this study. A total of 1297 variants were analyzed for the *UACA* locus by pooling patients from SBCSS and SBCS (15:70446893-71555932 in genome build 37).

### Statistical analysis

In the Pathways Study, we performed Cox proportional hazards regression for overall survival (OS) on each variant’s imputation dosage while controlling for age at diagnosis, body mass index, tumor grade, stage of disease, ER, progesterone receptor, and HER2 status, hormonal therapy, chemotherapy, radiation therapy, surgery type, and all population stratification principal components deemed significant by the Tracy-Widom statistic derived from EIGENSTRAT (Supplementary Table 1). The same covariates were included in the cox regression model when testing the association between recurrence/progression-free survival (PFS) and rs720251. Time to event was calculated from the date of diagnosis. The last date of follow-up for OS and breast cancer recurrence in the Pathways Study was 12/31/2017. The “survival” and “GWASTools” packages in R were used to perform single variant association analysis within each population for variants with MAF > 5% in the corresponding population. MR-MEGA was adopted for trans-ethnic meta-analysis across populations. To correct for genomic inflation, the standard errors of beta coefficients from the Cox models were multiplied by the square root of the genomic-inflation factor ($\lambda$) before running MR-MEGA when $\lambda \ge 1.06$. GWAS were also run in two separate patient groups: patients receiving Par-4 dependent chemotherapies versus the remaining patients. Specifically, the former group included patients who received doxorubicin as well as patients who were HER2+ and received HER2 targeted agents lapatinib, pertuzumab, or trastuzumab. In association analyses for death after recurrence and death without recurrence (all cause of death), as well as for analyses of breast-cancer specific death (BCSD) or death due to cardiovascular disease versus other cause of death, we used cumulative incidence functions to handle the two competing risks.

In the analysis of GERA cohort, reagent kit was added to the covariate list described above to control for possible effects of using two different Affymetrix reagent kits during genotyping. The last date of follow-up for OS in the GERA cohort was 9/30/2018.

Cox proportional hazards regression of OS was performed on variants of the *UACA* locus in the DBBR cohort and the SBCSS + SBCS cohort separately using the same model and covariates as described above in the analysis of the Pathways Study, including the principal components to control for population stratification. Principal components were not available for the association analysis in the DBBR cohort as only four variants were genotyped. The end of follow-up for OS in the DBBR cohort was 12/31/2019.

METASOFT was used in meta-analysis of the effect on OS of rs720251 across all four cohorts with Han and Eskin’s Random Effects model, which was optimized to detect associations under heterogeneity^28^. Beta coefficients from the Cox models and genomic inflation-corrected standard errors (described above in running MR-MEGA) were used as inputs for METASOFT. This model also calculates the posterior probability that an effect exists in each cohort as an *m*-value^44^. An *m*-value > 0.9 suggests existence of a genetic effect in that cohort. METASOFT was also used for meta-GWAS within ER− patients across Pathways European and African populations. ForestPMPlot was used to create the forest plot and the PM plot.

### Risk allele identification for GWAS study PMID:31904872

We download the accompanying genotyping and clinical data from https://figshare.com/articles/dataset/IJC_Clinical_features_and_SNPs_genotype_xlsx/11474403/1, and test association between the four GWAS variants and disease-free survival in ER+ patients while controlling for available covariates (tumor stage, hormone treatment, and chemotherapy status).

### Linkage disequilibrium calculation

Population-specific measures of linkage disequilibrium between SNP pairs were calculated using LDpair^45^ based on reference haplotypes from Phase 3 (Version 5) of the 1000 Genomes Project.

### Risk allele frequency comparison

Using non-parametric bootstrapping, we compared the risk allele (T) frequency of rs720251 in the GERA prevalent breast cancer patients taking Par-4 dependent chemotherapies ($F_R$) with its population frequency estimated from the GERA incident breast cancer patients ($F_P$). The null hypothesis is$H_0:F_R \ge F_P$, and the alternative hypothesis is $H_a:F_R < F_P$. We re-sampled the imputation dosage of rs720251 with replacement 10,000 times in the prevalent patients taking the particular treatment as well as in all incident patients and then calculated T allele frequency each time ($F_{R,i}$ and $F_{P,i}$, where $i = 1, \ldots ,10000$). The bootstrap *P*-value was calculated as $P = \mathop {\sum }\nolimits_{i = 1}^{10000} I(F_{R,i} \ge F_{P,i})/10000$. The same procedure was used to compare T allele frequency of rs720251 between the GERA prevalent patients without Par-4 dependent chemotherapies and all GERA incident patients. The analysis was repeated to use all European-descent patients from the Pathways Study to estimate T allele frequency at population level.

### eQTL analysis

cis-eQTL summary statistics from the eQTLGen consortium were download from the eQTLGen website (https://www.eqtlgen.org/cis-eqtls.html). cis-eQTLs with FDR < 0.05 were considered significant. The cis-eQTL analysis across tissues in Genotype-Tissue Expression (GTEx) Project were obtained from the GTEx Portal on 4/21/2020.

### Transcriptome-wide association study

Imputation dosages of variants with MAF > 5% and Rsq $\ge 0.8$ in the Pathways European population were used as input to PrediXcan^46^ with whole-blood prediction model trained in 922 whole-blood samples from Depression Genes and Networks (DGN)^47^. Predicted gene expression was tested for association with OS using Cox proportional hazards regression with same covariates as in the model of GWAS (see “Statistical analysis” section above). A total 11,520 genes were tested, yielding a transcriptome-wide significance cutoff $4.34 \times 10^{ - 6}$.

### Reporting summary

Further information on research design is available in the Nature Research Reporting Summary linked to this article.

## Supplementary information

Supplementary Tables and Figures

Reporting Summary

## Data Availability

Individual-level genotype and imputation data of the Pathways Study is available through dbGaP (accession number: phs001534.v1.p1).

## Code availability

We used R (version 3.4.1) extensively for data analysis and creating plots. Additional software used in the study included PLINK 1.9 https://www.cog-genomics.org/plink/, EIGENSOFT v6.1.4 https://www.hsph.harvard.edu/alkes-price/software/, MR-MEGA v0.1.5 https://genomics.ut.ee/en/mr-mega, METASOFT v2.0.1 http://genetics.cs.ucla.edu/meta_jemdoc/index.html, ForestPMPlot v1.0.2 http://genetics.cs.ucla.edu/meta_jemdoc/index.html, PrediXcan https://github.com/hakyimlab/PrediXcan.
