# Supplementary material for: UACA locus is associated with breast cancer chemoresistance and survival
Source: NPJ Breast Cancer. 2022 Mar 23;8:39. doi: 10.1038/s41523-022-00401-5 (PMC8943134; doi:10.1038/s41523-022-00401-5)
Supplement: Supplementary file 1 — Supplementary Tables and Figures [file 41523_2022_401_MOESM1_ESM.pdf]

Supplementary Table 1. Patient characteristics*.

Patient characteristics	N (%)									
	Pathways EUR (n=2801)		Pathways EAS (n=450)		Pathways HIS (n=392)		Pathways AFR (n=330)		GERA incident patients (n=880)	
Age at diagnosis, y										
Mean	61.46		53.02		54.28		56.57		68.61	
Range	[23, 94]		[24, 85]		[26, 91]		[29, 89]		[31, 89]	
BMI										
Mean	28.65		25.1		29.71		32.8		28.27	
Range	[15.05, 72.41]		[16.64, 45.96]		[18.16, 58.92]		[19.14, 62]		[16.97, 65.70]	
Missing	1		0		0		0		18	
ER status										
Positive	2407	85.93%	374	83.11%	325	82.91%	220	66.67%	752	85.45%
Negative	393	14.03%	76	16.89%	67	17.09%	109	33.03%	124	14.09%
Missing	1	0.04%	0	0.00%	0	0.00%	1	0.30%	4	0.45%
PR status										
Positive	1857	66.30%	292	64.89%	251	64.03%	148	44.85%	588	66.82%
Negative	941	33.60%	158	35.11%	141	35.97%	181	54.85%	284	32.27%
Missing	3	0.11%	0	0.00%	0	0.00%	1	0.30%	8	0.91%
HER2 status										
Positive	323	11.53%	77	17.11%	61	15.56%	46	13.94%	87	9.89%
Negative	2364	84.40%	353	78.44%	312	79.59%	273	82.73%	727	82.61%
Missing	114	4.07%	20	4.44%	19	4.85%	11	3.33%	66	7.50%
Hormonal therapy										
Yes	2132	76.12%	341	75.78%	294	75.00%	196	59.39%	685	77.84%
No	651	23.24%	109	24.22%	97	24.74%	129	39.09%	194	22.05%
Missing	18	0.64%	0	0.00%	1	0.26%	5	1.52%	1	0.11%
Chemo therapy										
Yes	1200	42.84%	248	55.11%	215	54.85%	189	57.27%	275	31.25%
No	1591	56.80%	201	44.67%	175	44.64%	141	42.73%	605	68.75%

Missing	10	0.36%	1	0.22%	2	0.51%	0	0.00%	0	0.00%
Radiation therapy										
Yes	1318	47.05%	145	32.22%	168	42.86%	129	39.09%	516	58.64%
No	1483	52.95%	305	67.78%	224	57.14%	201	60.91%	364	41.36%
Grade										
I	801	28.60%	117	26.00%	81	20.66%	58	17.58%	259	29.43%
II	1207	43.09%	191	42.44%	164	41.84%	132	40.00%	391	44.43%
III/IV	635	22.67%	121	26.89%	123	31.38%	124	37.58%	180	20.45%
Missing	158	5.64%	21	4.67%	24	6.12%	16	4.85%	50	5.68%
Stage										
I	1573	56.16%	245	54.44%	201	51.28%	154	46.67%	512	58.18%
II	924	32.99%	161	35.78%	138	35.20%	126	38.18%	262	29.77%
III/IV	304	10.85%	44	9.78%	53	13.52%	50	15.15%	101	11.48%
Missing	0	0.00%	0	0.00%	0	0.00%	0	0.00%	5	0.57%
Surgery type										
None	83	2.96%	14	3.11%	16	4.08%	14	4.24%	49	5.57%
Lumpectomy	1747	62.37%	209	46.44%	225	57.40%	206	62.42%	591	67.16%
Mastectomy	971	34.67%	227	50.44%	151	38.52%	110	33.33%	240	27.27%

[*]: Pathways EUR, EAS, HIS, AFR correspond to Pathways patients of European, East Asian, Hispanic, and African population respectively.

Supplementary Table 2. The sample size and genomic inflation factor for each GWAS of OS.

Cohort	Patient group	# of patients	(%)	# of events	Sample size*	Event size*	Lambda†
Pathways European population	All patients	2801		464	2511	404	1.0579
	Patients taking Par-4 dependent chemotherapies	949	(33.88%)	169	862	145	1.0885
	Remaining patients	1852	(66.12%)	295	1649	259	1.0939
Pathways East Asian population	All patients	450		41	408	38	1.1692
	Patients taking Par-4 dependent chemotherapies	195	(43.33%)	28	186	26	1.3469
	Remaining patients	255	(56.67%)	13	222	12	–‡
Pathways Hispanic population	All patients	392		53	349	44	1.2964
	Patients taking Par-4 dependent chemotherapies	168	(42.86%)	27	148	24	1.6372
	Remaining patients	224	(57.14%)	26	201	20	1.5533
Pathways African population	All patients	330		71	299	66	1.2762
	Patients taking Par-4 dependent chemotherapies	149	(45.15%)	44	137	40	1.6749
	Remaining patients	181	(54.85%)	27	162	26	1.6193
GERA incident cases	All patients	880		84	754	58	1.1245
	Patients taking Par-4 dependent chemotherapies	158	(17.95%)	17	138	13	1.8105
	Remaining patients	722	(82.05%)	67	660	53	1.1042

*: the actual numbers used in the cox model after missing values were excluded.

†: genomic inflation was corrected when lambda value was > 1.06.

‡: model fitting failed due to limited number of death.

Supplementary Table 3. The characteristics of patients received Par-4 dependent chemotherapies in the Pathways European population (EUR) and in the DBBR cohort respectively.

Patient characteristics	N (%)				
	Pathways EUR (n=949)		DBBR (n=451)		P^*
Age at diagnosis, y					**8.47E-14**
Mean	55.67		51.01		
Range	[24, 82]		[23, 82]		
BMI					0.85
Mean	28.9		28.83		
Range	[18, 66.4]		[16.9, 60.5]		
ER status					**1.22E-04**
Positive	702	73.97%	287	63.64%	
Negative	247	26.03%	163	36.14%	
Missing	0	0.00%	1	0.22%	
PR status					0.61
Positive	501	52.79%	245	54.32%	
Negative	447	47.10%	205	45.45%	
Missing	1	0.11%	1	0.22%	
HER2 status					0.47
Positive	248	26.13%	126	27.94%	
Negative	676	71.23%	311	68.96%	
Missing	25	2.63%	14	3.10%	
Hormonal therapy					**4.69E-02**
Yes	671	70.71%	295	65.41%	
No	274	28.87%	154	34.15%	
Missing	4	0.42%	2	0.44%	
Radiation therapy					**< 2.2E-16**
Yes	219	23.08%	372	82.48%	
No	730	76.92%	73	16.19%	
Missing	0	0.00%	6	1.33%	
Grade					**1.34E-05**
I	104	10.96%	34	7.54%	
II	398	41.94%	146	32.37%	
III/IV	389	40.99%	244	54.10%	
Missing	58	6.11%	27	5.99%	
Stage					0.16
I	209	22.02%	120	26.61%	
II	495	52.16%	218	48.34%	
III/IV	245	25.82%	113	25.06%	
Surgery type					**4.43E-03**
None	54	5.69%	12	2.66%	
Lumpectomy	446	47.00%	246	54.55%	
Mastectomy	449	47.31%	193	42.79%	

*:Student's *t*-test was used to compare the continuous variables between cohorts. Fisher's exact test and $\chi 2$ test were used to compare binary and multi-categorical variables between the two cohorts. P-values < 0.05 were in bold.

Supplemental Table 4. Association with OS in the DBBR cohort.

Variant	Chr	Position	Alleles		Association	
			Effect	Other	HR	P
rs11855431	15	71029751	C	T	1.19	0.56
rs6494889	15	70994283	A	G	1.13	0.67
rs28607477	15	70947138	C	T	1.14	0.66
rs720251	15	70938445	T	C	1.11	0.72

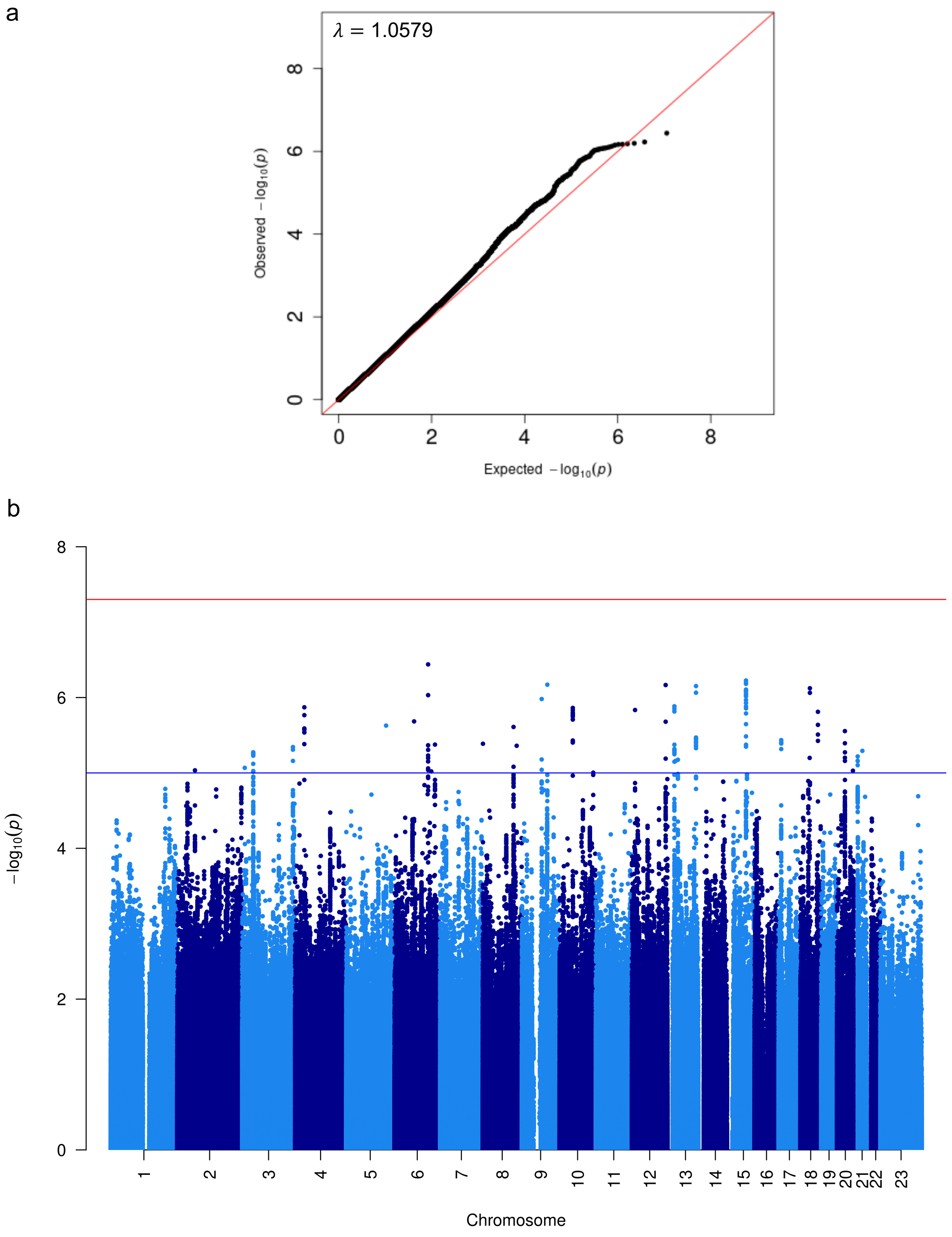

Supplementary Figure 1. QQ plot (a) and Manhattan plot (b) for GWAS of OS in the Pathways European population.

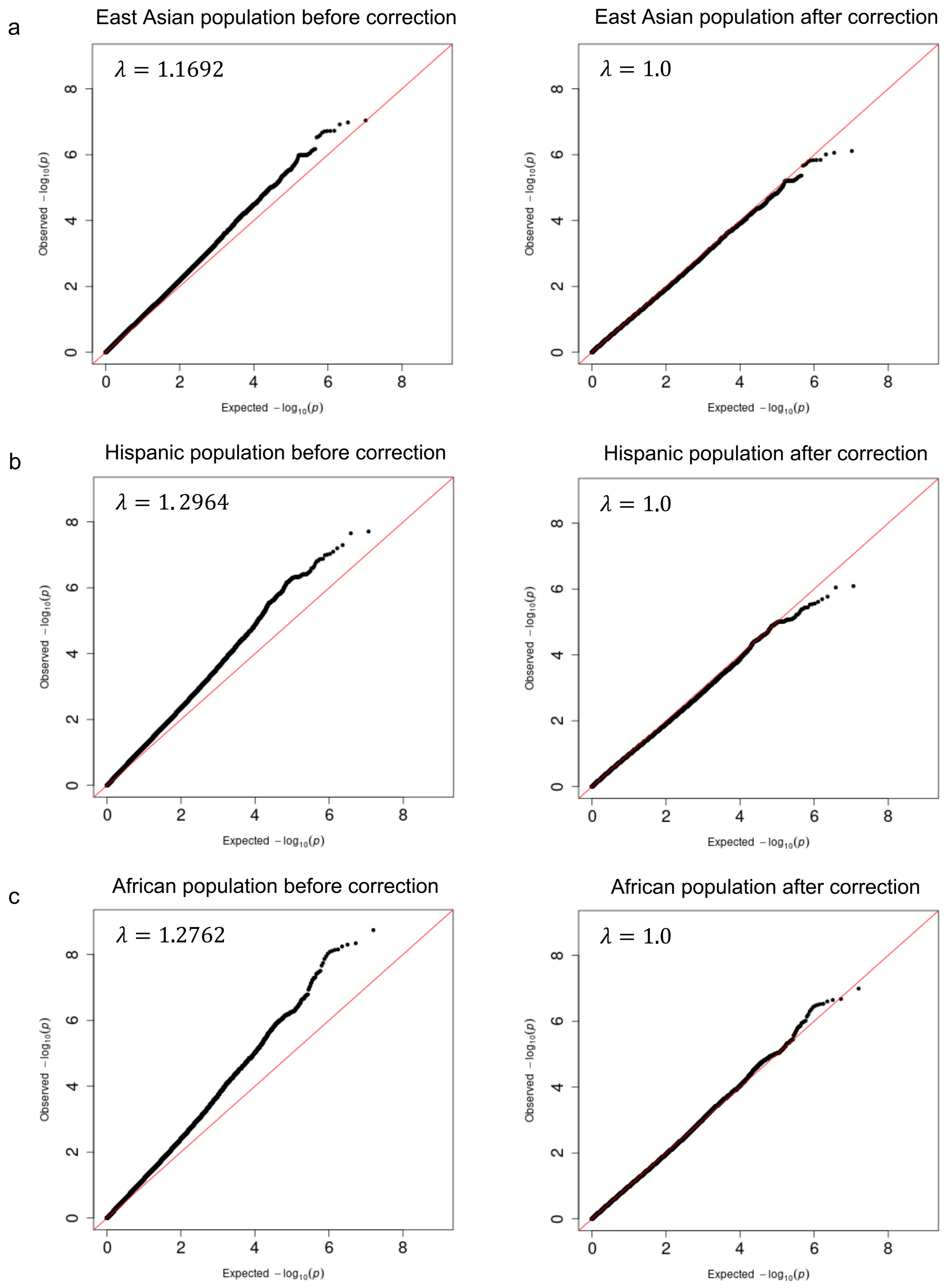

Supplementary Figure 2. QQ plot for GWAS of OS in the Pathways East Asian (a), Hispanic (b), and African (c) population respectively before and after correcting for genomic inflation.

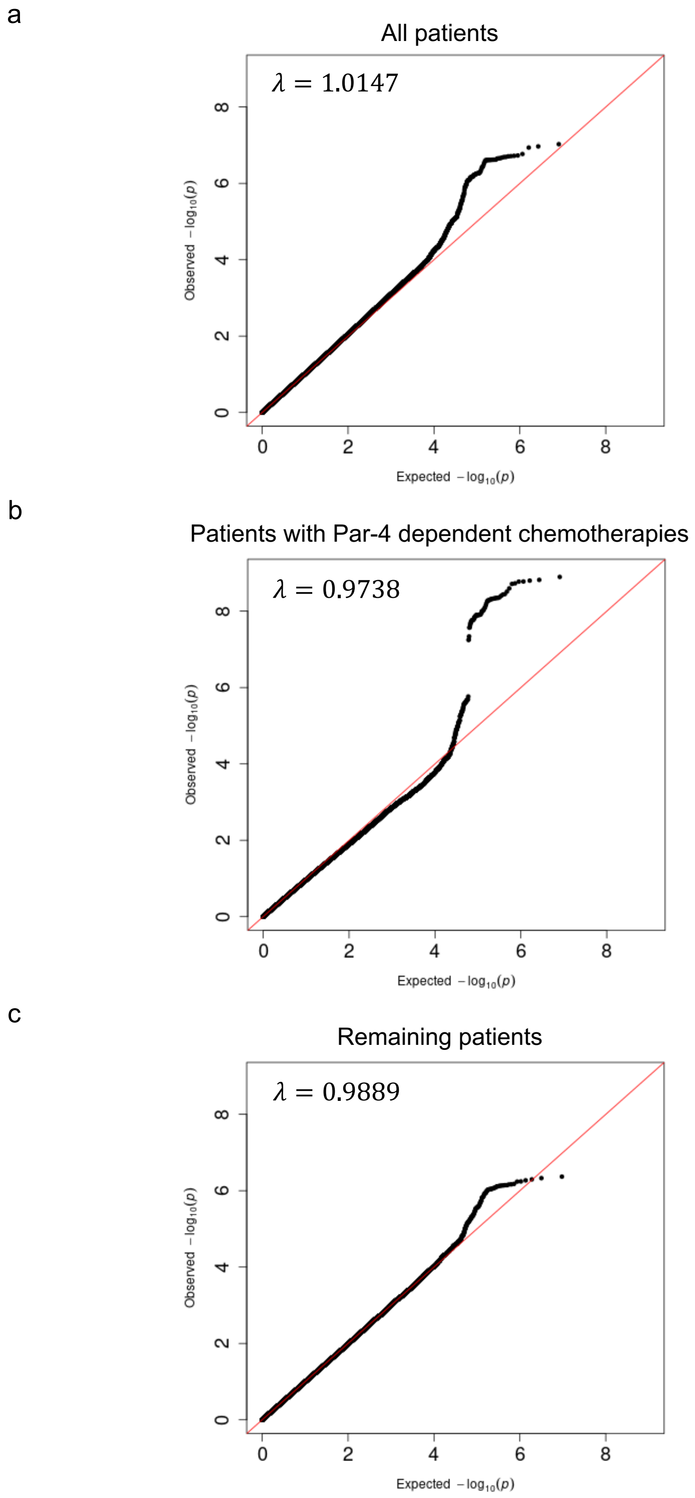

Supplementary Figure 3. QQ plot for trans-ethnic meta-GWAS of OS in the Pathways Study when including all patients (a), patients taking Par-4 dependent chemotherapies (b), and the remaining patients (c).

a

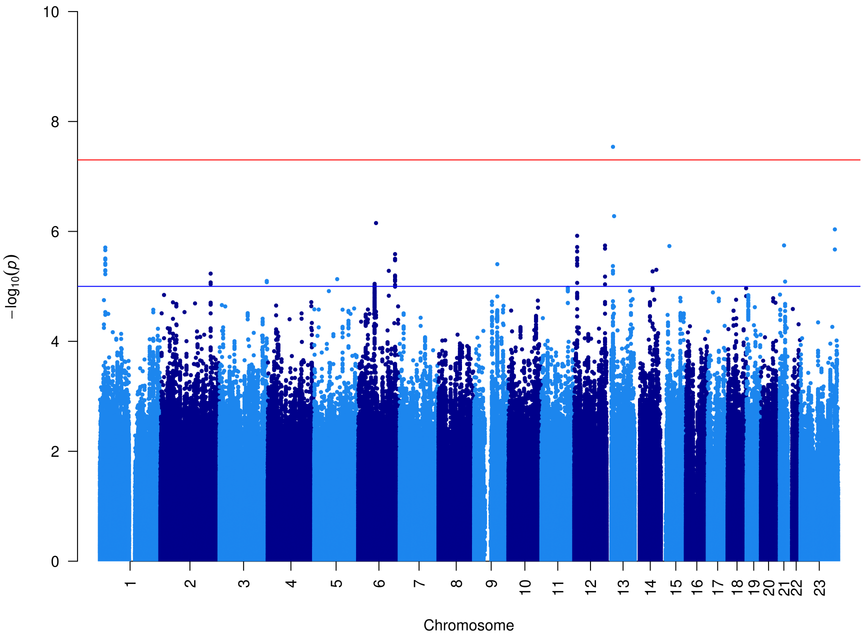

b

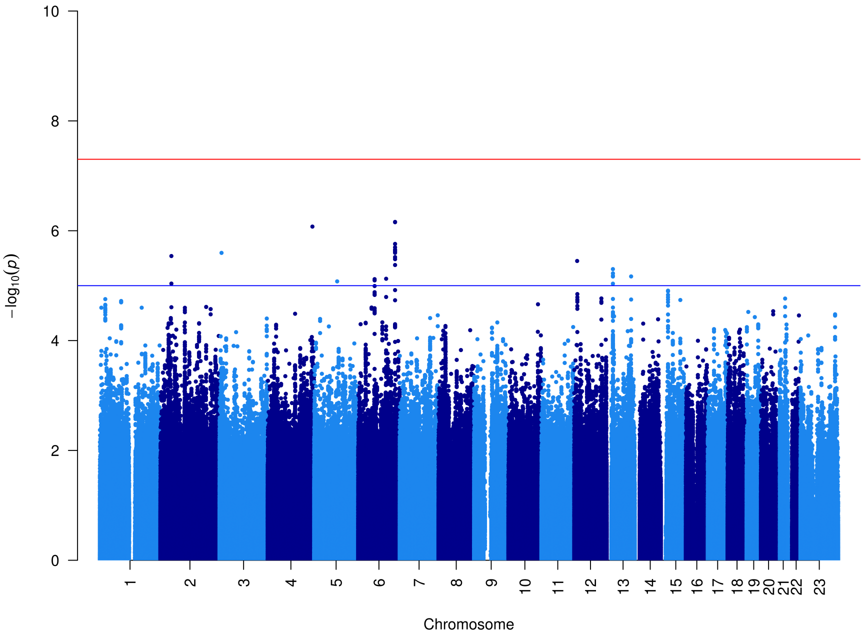

Supplementary Figure 4. Manhattan plot for GWAS of OS in the Pathways ER+ patients within European population (a), and across all four populations (b). The genomic inflation factor was 1.059 and 1.016 in the European-only GWAS and the trans-ethnic meta-GWAS respectively.

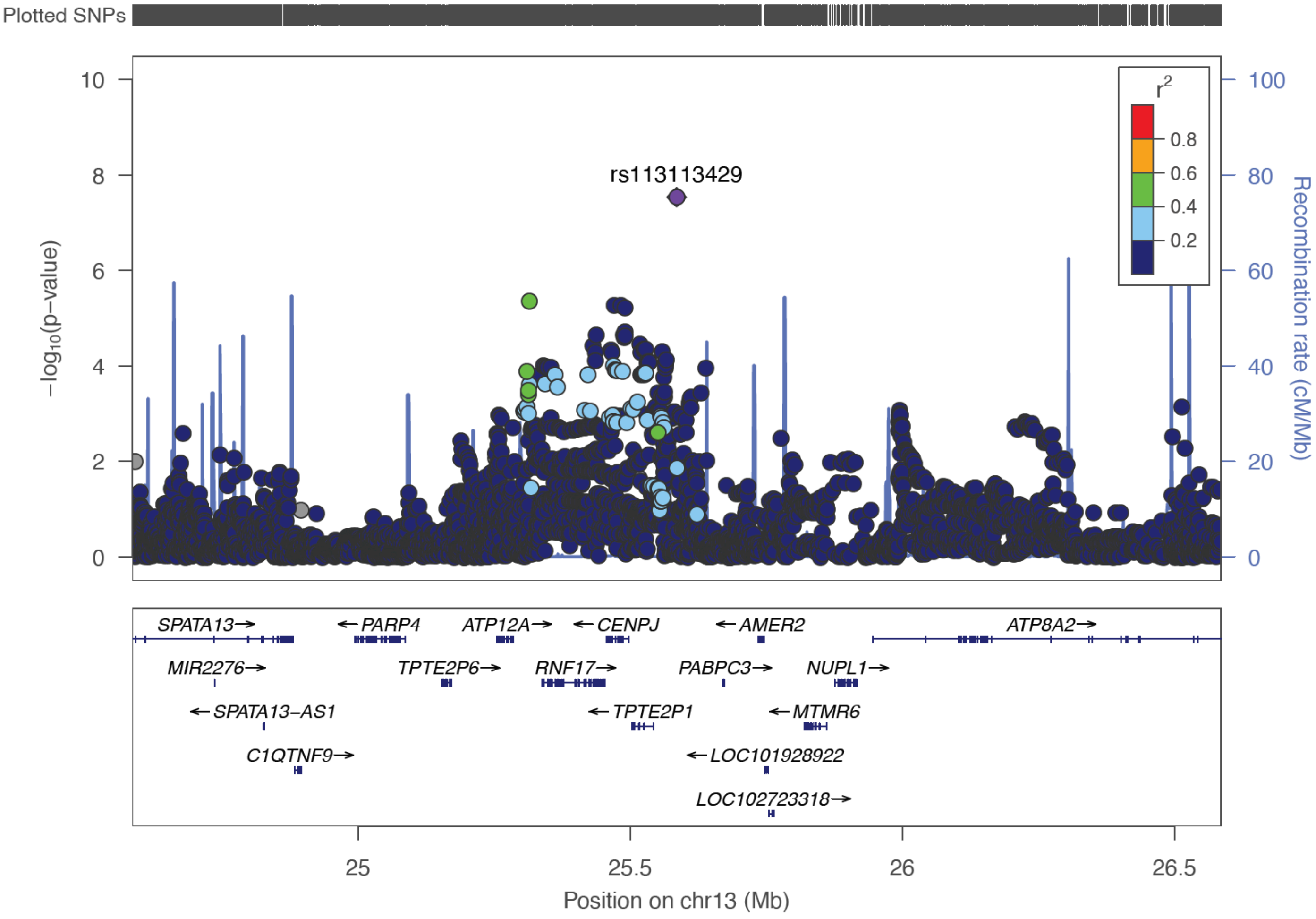

Supplementary Figure 5. Locuszoom plot for the top hit in GWAS of OS within the Pathways ER+ patients of European descent. The 2Mb region centered on the lead variant rs113113429 was plotted. LD structure was based on 1000 genomes EUR population (Nov 2014).

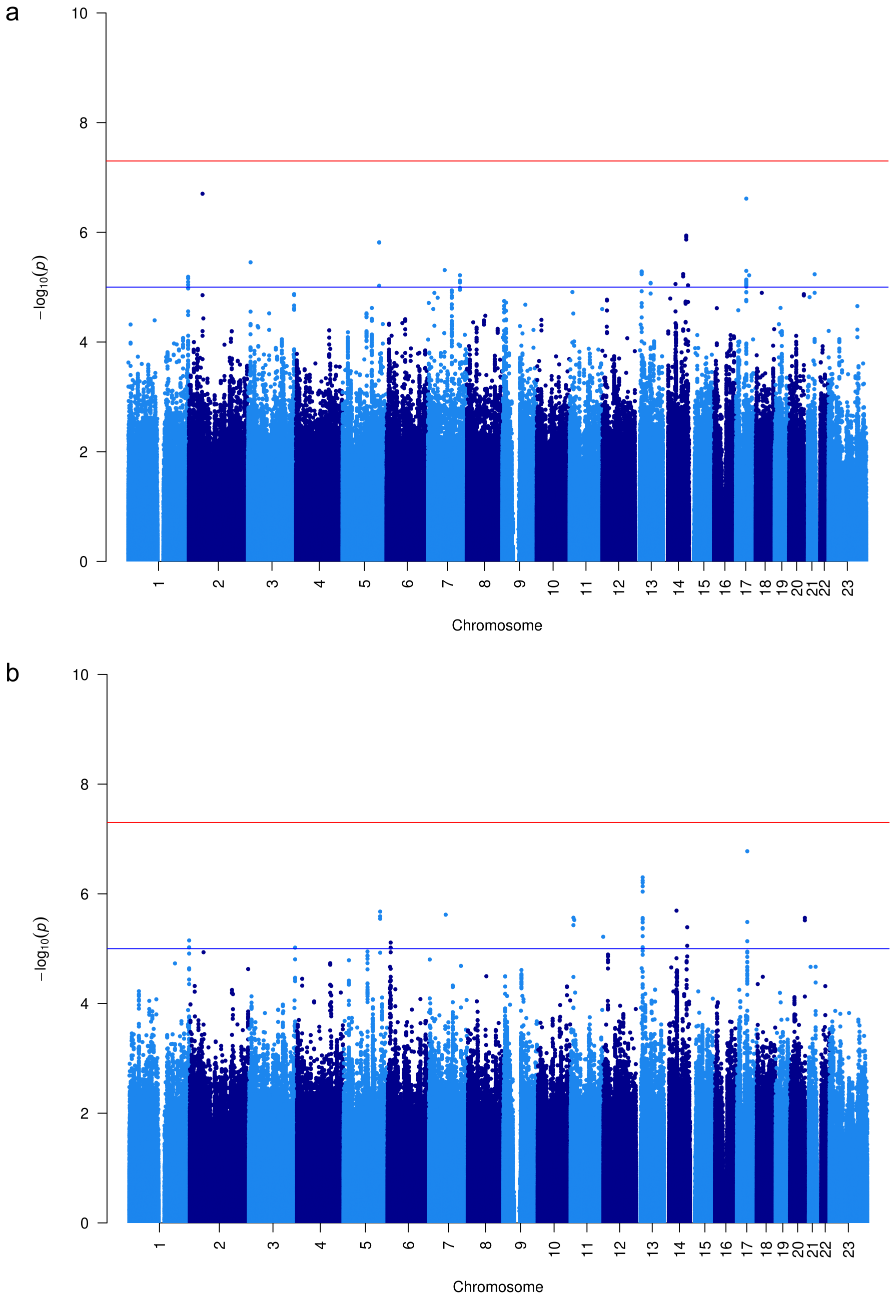

Supplementary Figure 6. Manhattan plot for GWAS of OS in the Pathways ER- patients within European population (a), and across populations (b). P-values in the European-only GWAS (a) were corrected for genomic inflation factor. Meta-GWAS for ER- patients (b) included only Pathways patients of European and African population because of too few events in the East Asian and Hispanic population (≤10). The genomic inflation factor for the meta-GWAS was 0.9820.

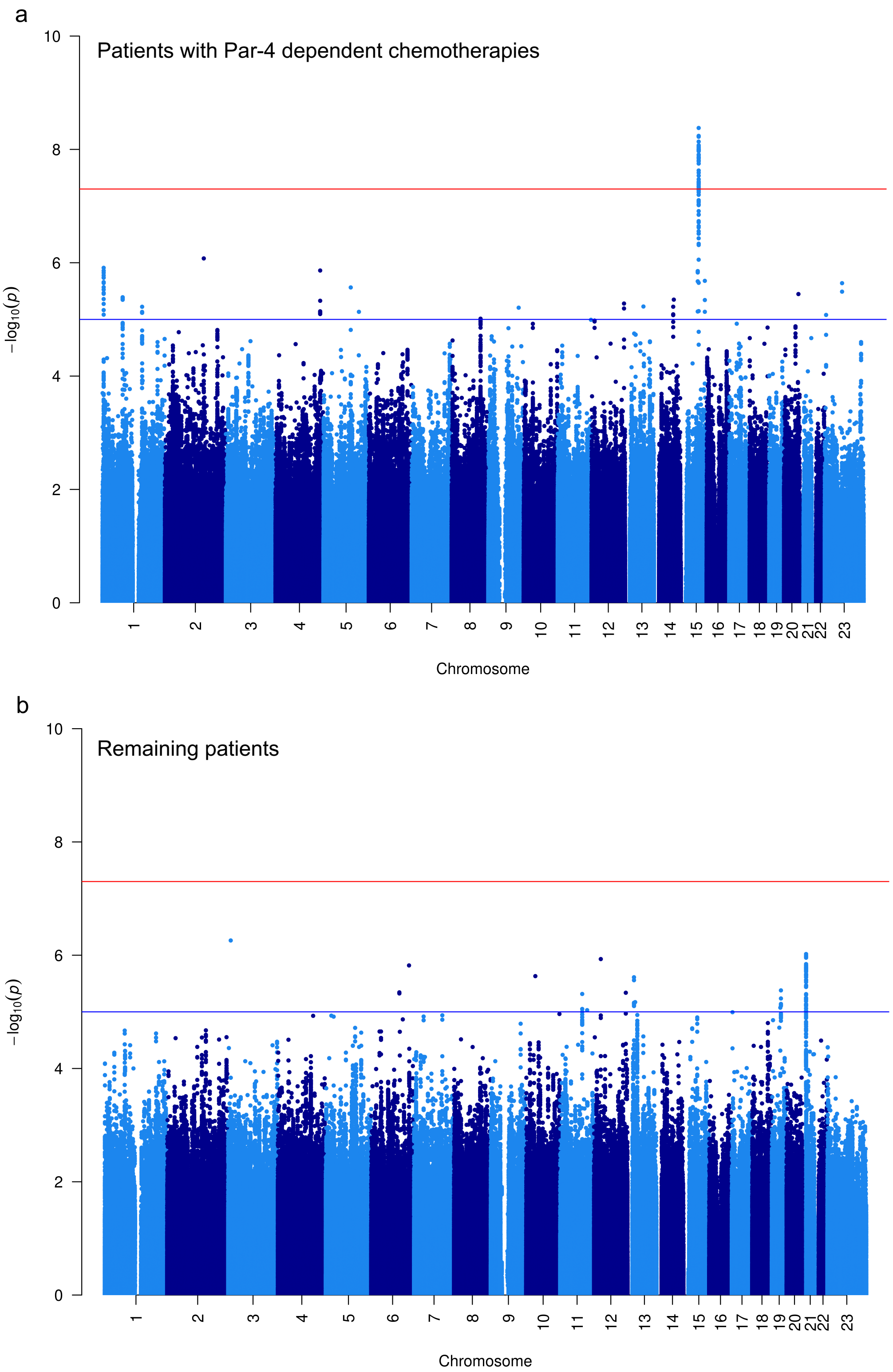

Supplementary Figure 7. Manhattan plot for GWAS of OS in the Pathways European population when including patients taking Par-4 dependent chemotherapies (a), and the remaining patients (b). P-values were corrected for genomic inflation.

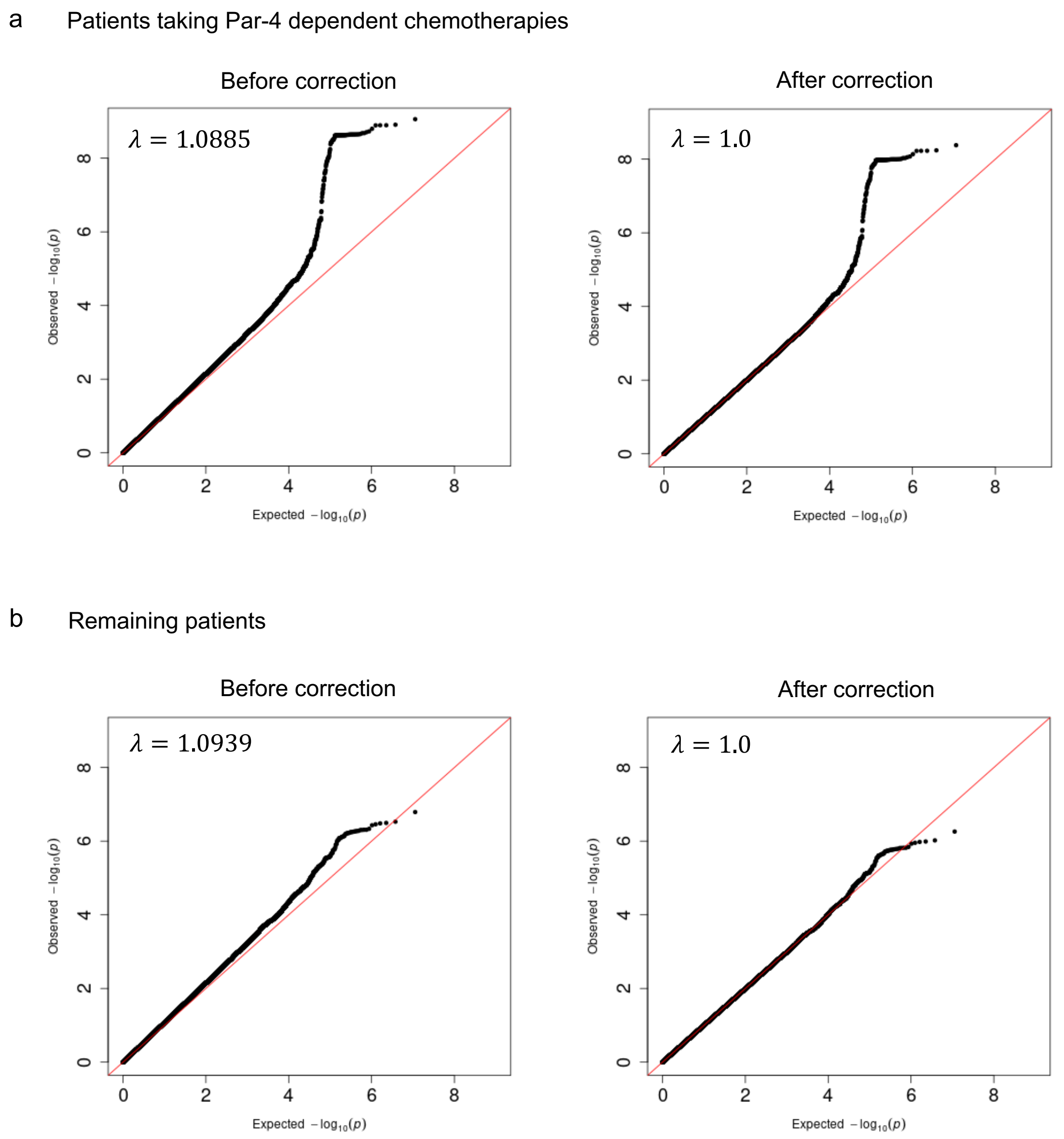

Supplementary Figure 8. QQ plot for GWAS of OS in the Pathways European population when including patients taking Par-4 dependent chemotherapies (a), and the remaining patients (b) respectively before and after correcting for genomic inflation.

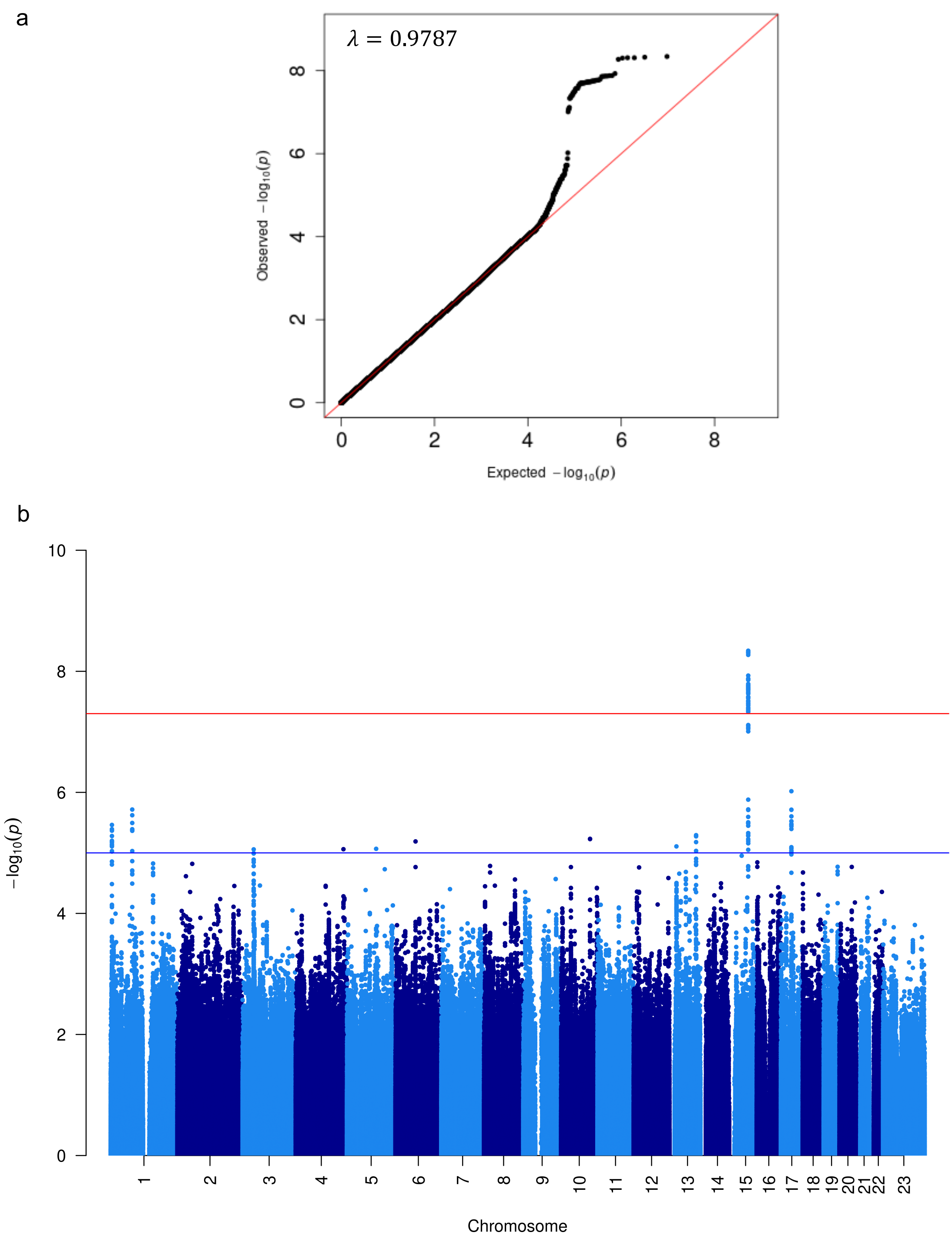

Supplementary Figure 9. QQ plot (a) and Manhattan plot (b) for trans-ethnic meta-GWAS of OS in the Pathways Study when only including patients taking Par-4-dependent chemotherapies. The East Asian population was excluded from this meta-analysis for a direct comparison with the meta-analysis of the remaining patients, where the result from East Asian population was not available due to small number of events.

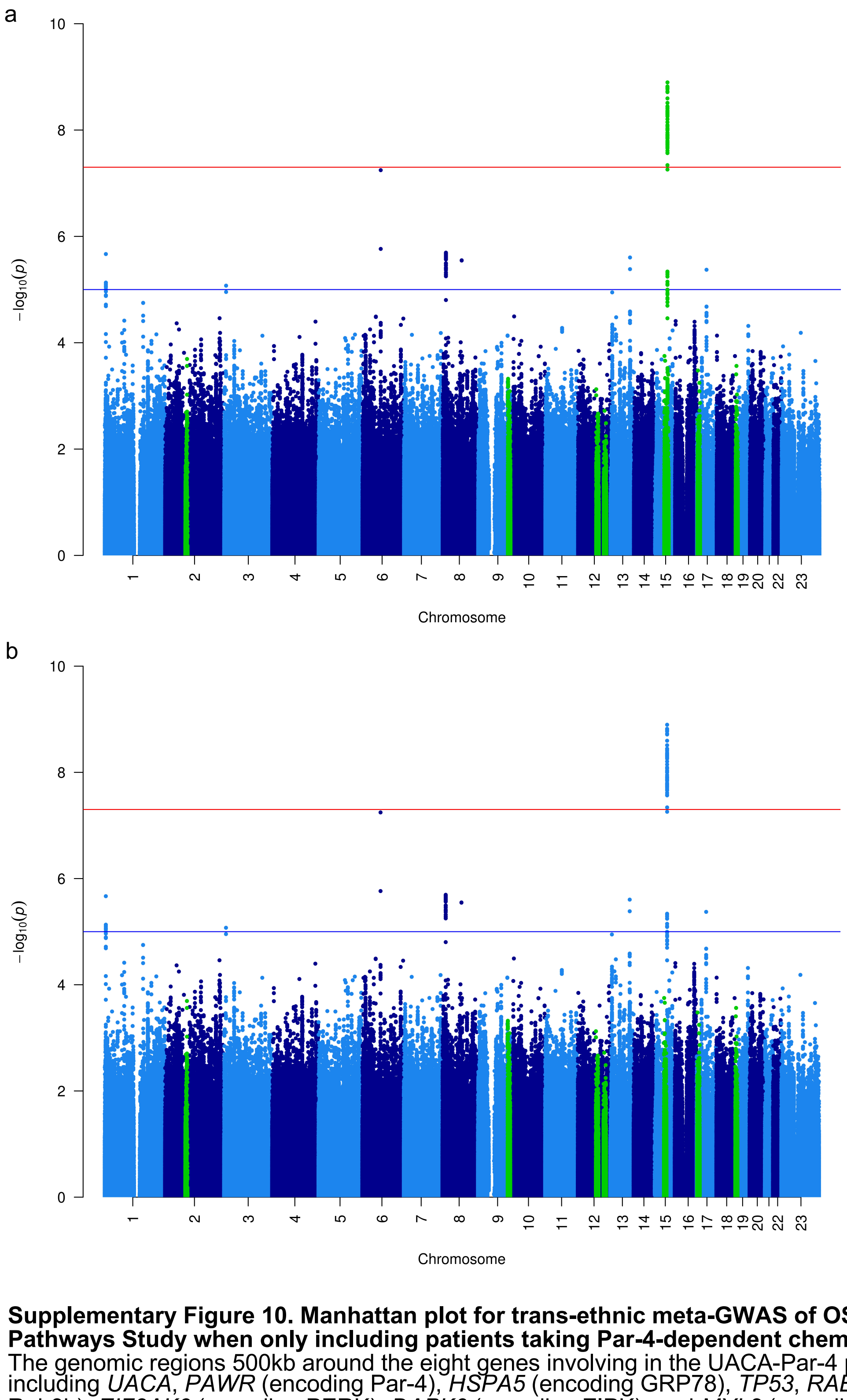

Supplementary Figure 10. Manhattan plot for trans-ethnic meta-GWAS of OS in the Pathways Study when only including patients taking Par-4-dependent chemotherapies. (a) The genomic regions 500kb around the eight genes involving in the UACA-Par-4 pathway[21,22,31,32], including *UACA*, *PAWR* (encoding Par-4), *HSPA5* (encoding GRP78), *TP53*, *RAB8B* (encoding Rab8b), *EIF2AK3* (encoding PERK), *DAPK3* (encoding ZIPK), and *MYL2* (encoding MLC2), were highlighted in green. (b) The same genomic regions except the *UACA* lous were highlighted in green.

Tissue	Samples	NES	p-value	m-value
Muscle - Skeletal	706	0.271	8.3e-13	1.00
Nerve - Tibial	532	0.244	1.4e-12	1.00
Heart - Left Ventricle	386	0.276	1.6e-10	1.00
Artery - Tibial	584	0.185	1.2e-9	1.00
Colon - Transverse	368	0.310	4.4e-9	1.00
Esophagus - Muscularis	465	0.200	6.4e-9	1.00
Artery - Aorta	387	0.214	1.1e-8	1.00
Adipose - Subcutaneous	581	0.201	6.3e-8	1.00
Adipose - Visceral (Omentum)	469	0.205	5.4e-7	1.00
Esophagus - Mucosa	497	0.167	6.5e-7	1.00
Esophagus - Gastroesophageal Junction	330	0.253	8.5e-7	1.00
Lung	515	0.142	1.5e-6	1.00
Stomach	324	0.249	4.0e-6	1.00
Pancreas	305	0.236	7.0e-6	1.00
Whole Blood	670	0.210	1.0e-5	1.00
Thyroid	574	0.155	1.3e-5	1.00
Heart - Atrial Appendage	372	0.204	7.1e-5	1.00
Testis	322	0.218	8.9e-5	1.00
Breast - Mammary Tissue	396	0.135	4.9e-4	1.00
Cells - Cultured fibroblasts	483	0.122	6.5e-4	1.00
Colon - Sigmoid	318	0.166	2.2e-3	0.992
Spleen	227	0.165	3.4e-3	0.992
Skin - Sun Exposed (Lower leg)	605	0.100	3.5e-3	0.952
Skin - Not Sun Exposed (Suprapubic)	517	0.110	3.6e-3	0.963
Ovary	167	0.229	0.02	0.992
Brain - Frontal Cortex (BA9)	175	0.124	0.02	0.985
Brain - Amygdala	129	0.202	0.02	0.998
Small Intestine - Terminal Ileum	174	0.152	0.03	0.973
Brain - Hippocampus	165	0.130	0.03	0.971
Adrenal Gland	233	0.134	0.05	0.987
Minor Salivary Gland	144	0.195	0.05	0.968
Brain - Nucleus accumbens (basal ganglia)	202	0.103	0.06	0.911
Artery - Coronary	213	0.0996	0.1	0.909
Brain - Substantia nigra	114	0.154	0.1	0.888
Brain - Cerebellum	209	0.0784	0.2	0.739
Pituitary	237	0.0682	0.2	0.680
Brain - Spinal cord (cervical c-1)	126	0.119	0.3	0.946
Brain - Hypothalamus	170	0.0632	0.3	0.726
Brain - Putamen (basal ganglia)	170	0.0669	0.3	0.770
Liver	208	0.0702	0.4	0.837
Brain - Cortex	205	-0.0372	0.5	0.00900
Uterus	129	0.0663	0.5	0.752
Vagina	141	0.0564	0.6	0.717
Prostate	221	0.0532	0.6	0.830
Kidney - Cortex	73	0.0616	0.6	0.809
Brain - Cerebellar Hemisphere	175	0.0278	0.7	0.582
Cells - EBV-transformed lymphocytes	147	-0.0422	0.8	0.685
Brain - Anterior cingulate cortex (BA24)	147	-0.0185	0.8	0.279
Brain - Caudate (basal ganglia)	194	0.00411	0.9	0.0300

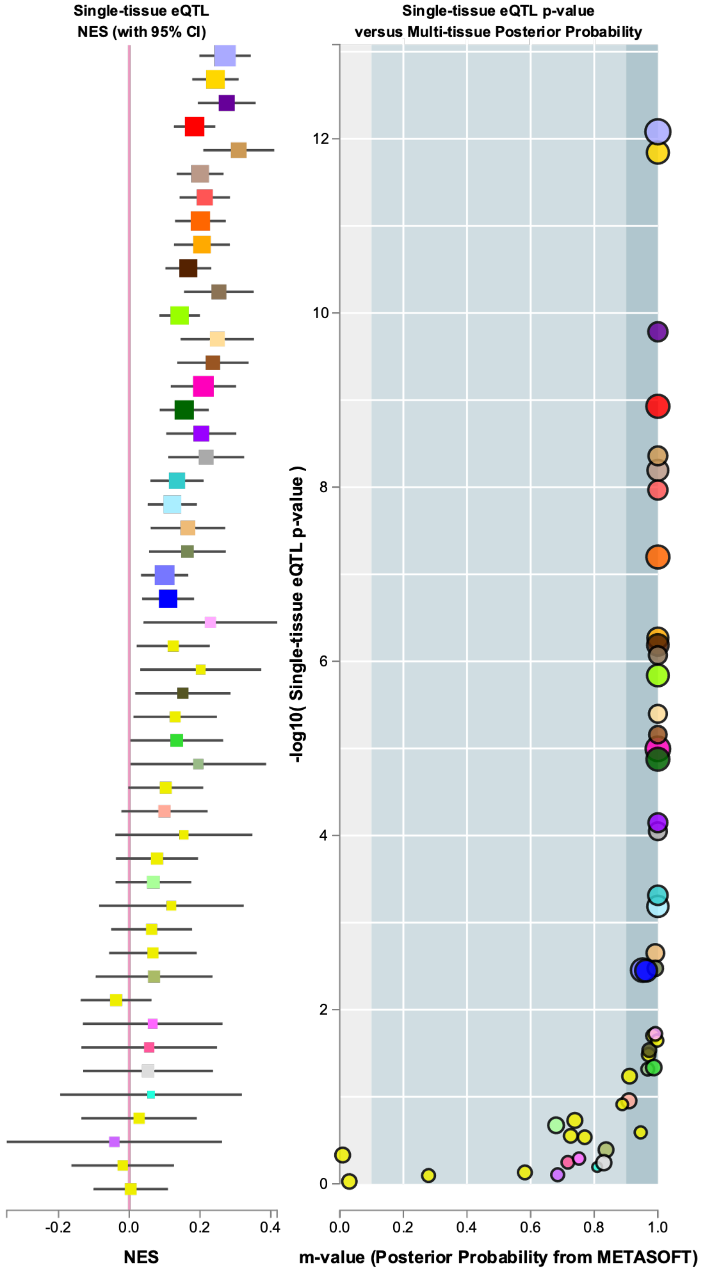

Supplementary Figure 11. Multi-tissue eQTL Comparison for *UACA* (ENSG00000137831.14) and rs62019060. Meta-Analysis Random Effect Model2 $P = 3.70{\times}10^{-124}$. The 17 tissues where rs62019060 was found as an significant eQTL for *UACA* were highlighted in red rectangles. The normalized effect size (NES) of the eQTLs is defined as the slope of the linear regression of normalized expression data versus the three genotype categories using single-tissue eQTL analysis, representing eQTL effect size. It is computed as the effect of the alternative allele (ALT) relative to the reference allele (REF) in the human genome reference GRCh38/hg38. NES are computed in a normalized space where magnitude has no direct biological interpretation. p-value is from a t-test that compares observed NES from single-tissue eQTL analysis to a null NES of 0. m-value is the posterior probability that an eQTL effect exists in each tissue tested in the cross-tissue meta-analysis. The m-value ranges between 0 and 1. (Data Source: GTEx Analysis Release V8, dbGaP Accession phs000424.v8.p2)

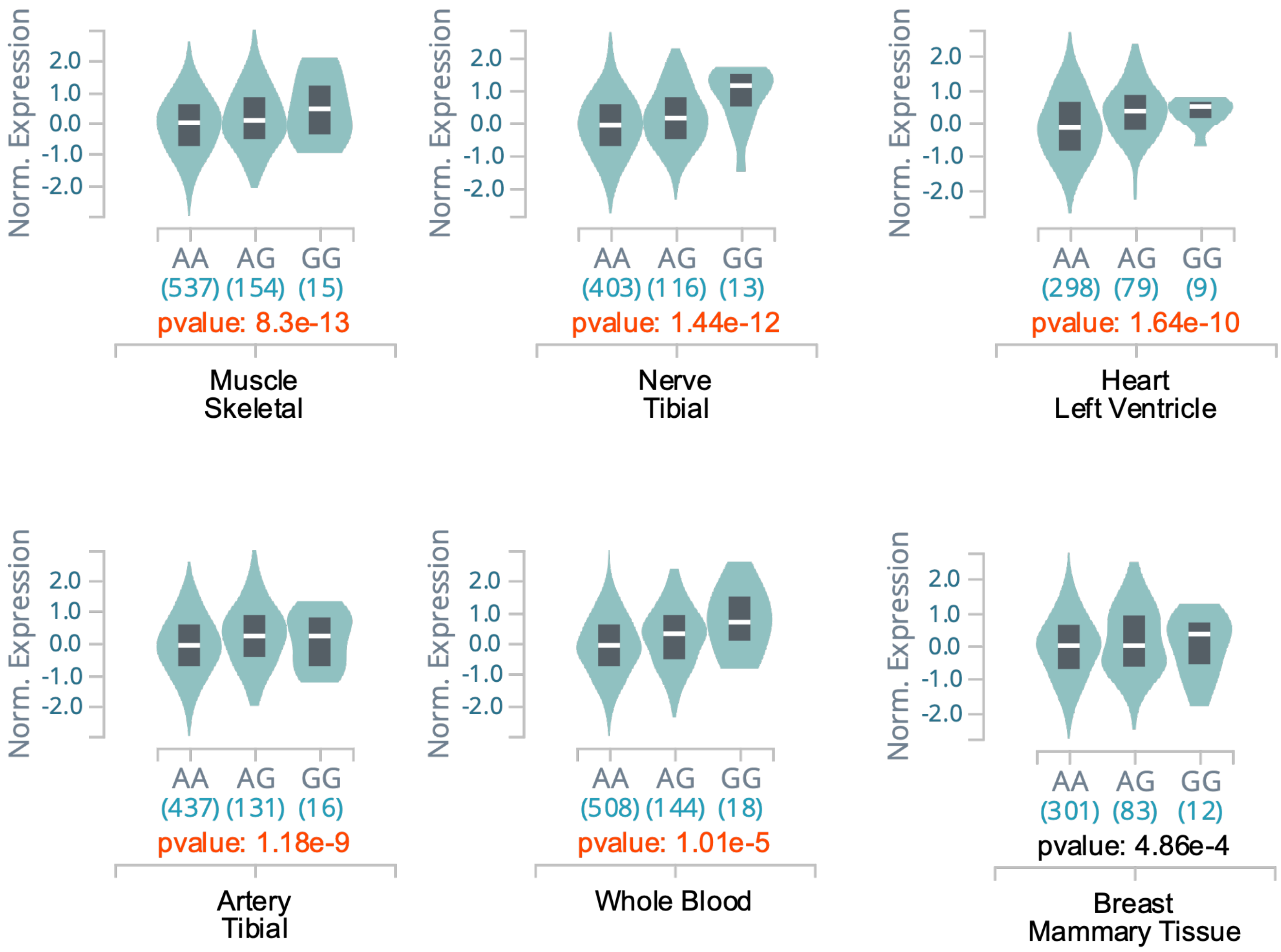

Supplementary Figure 12. eQTL violin plots for *UACA* (ENSG00000137831.14) and rs62019060. *UACA* expression in the four tissues with the lowest eQTL p-values (Supplementary Figure 11) plus whole blood and breast mammary tissue were plotted. Significant eQTL p-values were highlighted in red. (Data Source: GTEx Analysis Release V8, dbGaP Accession phs000424.v8.p2)
