# Supplementary material for: UACA locus is associated with breast cancer chemoresistance and survival
Source: NPJ Breast Cancer. 2022 Mar 23;8:39. doi: 10.1038/s41523-022-00401-5 (PMC8943134; doi:10.1038/s41523-022-00401-5)
Supplement: Supplementary file 2 — Reporting Summary [file 41523_2022_401_MOESM2_ESM.pdf]

nature portfolio

Corresponding author(s): Qianqian Zhu
Last updated by author(s): Dec 8, 2021

Reporting Summary

Nature Portfolio wishes to improve the reproducibility of the work that we publish. This form provides structure for consistency and transparency in reporting. For further information on Nature Portfolio policies, see our Editorial Policies and the Editorial Policy Checklist.

Statistics

For all statistical analyses, confirm that the following items are present in the figure legend, table legend, main text, or Methods section.

n/a	Confirmed	
☐	☒	The exact sample size (*n*) for each experimental group/condition, given as a discrete number and unit of measurement
☐	☒	A statement on whether measurements were taken from distinct samples or whether the same sample was measured repeatedly
☐	☒	The statistical test(s) used AND whether they are one- or two-sided<br>*Only common tests should be described solely by name; describe more complex techniques in the Methods section.*
☐	☒	A description of all covariates tested
☐	☒	A description of any assumptions or corrections, such as tests of normality and adjustment for multiple comparisons
☐	☒	A full description of the statistical parameters including central tendency (e.g. means) or other basic estimates (e.g. regression coefficient) AND variation (e.g. standard deviation) or associated estimates of uncertainty (e.g. confidence intervals)
☐	☒	For null hypothesis testing, the test statistic (e.g. *F*, *t*, *r*) with confidence intervals, effect sizes, degrees of freedom and *P* value noted<br>*Give P values as exact values whenever suitable.*
☒	☐	For Bayesian analysis, information on the choice of priors and Markov chain Monte Carlo settings
☒	☐	For hierarchical and complex designs, identification of the appropriate level for tests and full reporting of outcomes
☐	☒	Estimates of effect sizes (e.g. Cohen's *d*, Pearson's *r*), indicating how they were calculated

Our web collection on statistics for biologists contains articles on many of the points above.

Software and code

Policy information about availability of computer code

Data collection	*Provide a description of all commercial, open source and custom code used to collect the data in this study, specifying the version used OR state that no software was used.*
Data analysis	We used R (version 3.4.1) extensively for data analysis and creating plots. Additional software used in the study included PLINK 1.9 https://www.cog-genomics.org/plink/, EIGENSOFT v6.1.4 https://www.hsph.harvard.edu/alkes-price/software/, MR-MEGA v0.1.5 https://genomics.ut.ee/en/mr-mega, METASOFT v2.0.1 http://genetics.cs.ucla.edu/meta_jemdoc/index.html, ForestPMPlot v1.0.2 http://genetics.cs.ucla.edu/meta_jemdoc/index.html, PrediXcan https://github.com/hakyimlab/PrediXcan.

For manuscripts utilizing custom algorithms or software that are central to the research but not yet described in published literature, software must be made available to editors and reviewers. We strongly encourage code deposition in a community repository (e.g. GitHub). See the Nature Portfolio guidelines for submitting code & software for further information.

Data

Policy information about availability of data

All manuscripts must include a data availability statement. This statement should provide the following information, where applicable:

- Accession codes, unique identifiers, or web links for publicly available datasets
- A description of any restrictions on data availability
- For clinical datasets or third party data, please ensure that the statement adheres to our policy

Individual-level genotype and imputation data of the Pathways Study is available through dbGaP (accession number: phs001534.v1.p1).

Field-specific reporting

Please select the one below that is the best fit for your research. If you are not sure, read the appropriate sections before making your selection.

☒ Life sciences ☐ Behavioural & social sciences ☐ Ecological, evolutionary & environmental sciences

For a reference copy of the document with all sections, see nature.com/documents/nr-reporting-summary-flat.pdf

Life sciences study design

All studies must disclose on these points even when the disclosure is negative.

Sample size	Sample sizes are reported in the article and in Supplementary Table 1 and 2.
Data exclusions	Genotyped samples failed quality control were excluded from analysis. Variants failed quality control or had low imputation quality (Rsq < 0.3) were excluded from analysis.
Replication	We attempted to replicate our finding in three independent breast cancer survivor cohorts: the Genetic Epidemiology Research on Aging (GERA) Cohort, the Data Bank and Biorepository (DBBR) cohort, and the Shanghai Breast Cancer Survival Study (SBCSS) and Shanghai Breast Cancer Study (SBCS). The lead variant in patients of European population in the Pathways Study, rs720251, associated with OS in the SBCSS and SBCS patients treated with anthracyclines (P=1.29×10^(-4)). The GERA breast cancer cohort included both a prospective component, which involved 880 incident cases who had breast cancer diagnosis after collection of biospecimens for genotyping, and a retrospective component, which involved 1,983 prevalent cases who had cancer diagnosis before biospecimen collection. Of the 880 incident cases, 158 (18%) received anti-HER2 or doxorubicin therapies. We observed a non-significant yet consistent trend that UACA locus was associated with OS among the 158 patients treated with the Par4-dependent agents (HR=1.61, P=0.67 for the minor allele T of rs720251). Consistent with our finding, we observed that the prevalent cases who were treated with Par4-dependent agents represented a survival bias and carried T alleles at a lower frequency than that in the incident cases (P=5.50×10^(-3)). The association between OS and rs720251 was not significant in the DBBR cohort (P=0.72). In comparison to the Pathways Study, the DBBR cohort were younger (mean age at diagnosis: 51 vs. 55.7 years) and more likely to receive radiation therapy (82.5% vs. 23.1%).
Randomization	not applicable
Blinding	not applicable

Reporting for specific materials, systems and methods

We require information from authors about some types of materials, experimental systems and methods used in many studies. Here, indicate whether each material, system or method listed is relevant to your study. If you are not sure if a list item applies to your research, read the appropriate section before selecting a response.

Materials & experimental systems

n/a	Involved in the study	
☒	☐	Antibodies
☒	☐	Eukaryotic cell lines
☒	☐	Palaeontology and archaeology
☒	☐	Animals and other organisms
☐	☒	Human research participants
☒	☐	Clinical data
☒	☐	Dual use research of concern

Methods

n/a	Involved in the study	
☒	☐	ChIP-seq
☒	☐	Flow cytometry
☒	☐	MRI-based neuroimaging

Human research participants

Policy information about studies involving human research participants

Population characteristics	The Pathways Study is a prospective cohort study of a diverse population of recently diagnosed breast cancer survivors in Kaiser Permanente Northern California (KPNC). Recruitment into the cohort was from January 2006 to May 2013. Patient characteristics were provided in Supplementary Table 1.
Recruitment	Eligibility criteria were: age ≥ 21 years; current KPNC member; recently diagnosed with invasive breast cancer; no prior history of other invasive cancer other than non-melanoma skin cancer; primary language of English, Spanish, Cantonese, or Mandarin; and lived within a 65-mile radius of a field interviewer.
Ethics oversight	All study participants provided written informed consent before participating in the study and the Institutional Review Boards of all institutes involved approved the study protocols.

Note that full information on the approval of the study protocol must also be provided in the manuscript.
